# Whole-genome sequencing reveals divergent and shared selection signatures of heat stress adaptation in indigenous Ethiopian zebu cattle from dry-hot and humid-hot environments

**DOI:** 10.1371/journal.pone.0343484

**Published:** 2026-09-03

**Authors:** Endashaw Terefe, Gurja Belay, Abdulfatai Tijjani, Marcos Vinicius Barbosa da Silva, Jianlin Han, Bashir Salim, Olivier Hanotte

**Affiliations:** 1 Department of Animal Science, College of Agriculture and Environmental Sciences, Arsi University, Asella, Oromia, Ethiopia; 2 Department of Microbial Sciences and Genetics, College of Natural and Computational Sciences, Addis Ababa University, Addis Ababa, Ethiopia; 3 Feinstein Institute for Medical Research, Northwell Health, New Hyde Park, New York, United States of America; 4 Embrapa Dairy Cattle, Brazilian Agricultural Research Corporation (EMBRAPA), Juiz de Fora, Brazil; 5 Yazhouwan National Laboratory, Sanya, China; 6 Camel Research Center, King Faisal University, Al-Ahsa, Saudi Arabia; 7 International Livestock Research Institute (ILRI), Addis Ababa, Ethiopia; 8 Centre for Tropical Livestock Genetics and Health (CTLGH), The Roslin Institute, the University of Edinburgh, Midlothian, United Kingdom; 9 School of Life Sciences, University of Nottingham, University Park, Nottingham, United Kingdom; Sri Venkateswara Veterinary University, INDIA

## Abstract

African zebu cattle (*Bos indicus*) exhibit remarkable adaptations to extreme thermal conditions, yet the genomic basis of this resilience remains incompletely characterized. Ethiopia provides a unique natural setting in which closely related zebu populations have adapted divergently to dry-hot (DHETZ) and humid-hot (HHETZ) climates. In this study, we reanalyzed publicly available whole-genome sequencing datasets from 46 Ethiopian zebu cattle from five populations and compared them with Asian zebu, Sudanese zebu, African taurine, and European taurine breeds. By integrating genome-wide SNP analysis, population genetic structure assessment, and multiple selection scans (iHS, Hp, XP-EHH, and XP-CLR), we identified distinct and shared selection signatures between DHETZ and HHETZ. We detected 33.7 million and 34.2 million biallelic autosomal SNPs in DHETZ and HHETZ, respectively. Ethiopian zebu clustered closely with Sudanese zebu but showed clear divergence from Asian zebu and taurine breeds. DHETZ and HHETZ exhibited very low genetic differentiation (F_ST_ = 0.0063), consistent with their shared ancestry; however, each group displayed unique selection signals. In DHETZ, iHS and Hp detected 298 and 113 candidate regions, respectively, whereas in HHETZ, they detected 244 and 138 regions, respectively. Cross-population XP-EHH and XP-CLR analyses identified 163 and 227 divergent regions between DHETZ and HHETZ, respectively. Integration of the four selection scans identified 19 high-confidence candidate regions in DHETZ and 13 in HHETZ. DHETZ showed strong selection in genes involved in oxidative stress regulation, protein folding, mitochondrial function, and vascular remodeling, including *SESN2*, *DNAJC8*, *GRPEL2*, *ABLIM3*, and *AFAP1L1*. In contrast, HHETZ displayed signatures in genes associated with immune responses, energy metabolism, and angiogenesis inhibition, including *MYD88*, *PRKACA*, *PRKACB*, and *WIF1*. Several genes, including *VEGFC*, *TNIP3*, and *DMXL2*, were under selection in both groups, suggesting conserved mechanisms of thermotolerance and reproductive adaptation. The shared *VEGFC* signal and the HHETZ-specific *WIF1* signal may indicate a distinct vascular regulatory mechanism in the dry-hot and humid-hot environments. Our results reveal a dual pattern of genomic adaptation in Ethiopian zebu cattle and provide candidate loci for future validation and climate-resilient livestock breeding.

## Introduction

Africa harbors one of the most genetically diverse and phenotypically distinct cattle populations, broadly classified into African zebu (*Bos indicus*), Sanga (taurine-zebu hybrids), Zenga (Sanga-zebu hybrids), and African taurine (*Bos taurus*) breeds [1,2]. Among these, African zebu cattle are the most abundant and widely distributed, particularly across arid and semi-arid regions [3,4]. These cattle exhibit distinct morphological and physiological adaptations to extreme environments, including a prominent hump, large ears, loose skin, and variable coat colors [5,6]. Such features enhance thermoregulation and water conservation, rendering zebu cattle highly suited to tropical and arid climates [7].

Whole-genome studies have revealed substantial genetic diversity within African cattle, reflecting their adaptation to diverse agro-climatic conditions [8,9]. The complex admixture history of African cattle, particularly in the Horn of Africa, has shaped their genomic landscape, supporting resilience to heat stress, disease pressure, and nutritional challenges [8,10,11]. Environmental stressors such as pathogens, high temperatures, and altitudes have driven positive selection on functional genomic regions, enhancing survival in harsh conditions [12]. Indeed, heat stress adaptation in tropical cattle involves an integrated suite of morphological, behavioral, physiological, neuroendocrine, and metabolic modifications [13,14]. For example, increased sweating capacity enhances heat dissipation, while lighter coat color, shorter hair, and reduced body size improve thermotolerance [13,15,16].

At the genomic level, candidate genes associated with heat adaptation have been previously identified in African and other cattle populations [17,18]. These previously identified candidate genes are linked to oxidative stress regulation (*SOD1*, *GPX7*, and *PLCB1*), sweat gland development, hair-coat modification, and energy homeostasis [15,19]. Heat shock proteins (HSPs) such as *HSF5*, *HSPA9,* and and *DNAJC18* stabilize protein function under thermal stress, while the endocrine system, through corticotropin-releasing hormone, adrenocorticotropic hormone, and antidiuretic hormone, regulates thermoregulation, water balance, and metabolic adjustments [20,21]. Notably, the *PRLH* gene and its receptor, *PRLR,* have been linked with the slick hair phenotype, which enhances heat dissipation [13,22]. These physiological responses are expected to vary according to the type of thermal stress experienced by animals. Dry-hot environments are characterized by high ambient temperature, low humidity, limited water availability, and strong solar radiation, whereas humid-hot environments combine elevated temperature with high humidity, reducing the efficiency of evaporative cooling but often increasing pathogen and vector pressure. Therefore, cattle populations inhabiting these contrasting environments may rely on both shared thermotolerance mechanisms and environment-specific adaptive pathways.

Ethiopia represents a unique ecological setting where zebu cattle are found in close geographic proximity, yet they are adapted to contrasting environments, ranging from dry-hot (arid) lowlands to humid-cold highlands [11,12]. Indigenous Ethiopian cattle are predominantly indicine (*B. taurus indicus*), except for the admixed Sheko breed, which carries the highest taurine ancestry among Ethiopian cattle populations [23]. Despite adaptation to divergent environments, Ethiopian zebu exhibit high genetic relatedness, making them an excellent model for identifying genomic regions under selection for thermotolerance [11,24]. Although previous work, including Terefe et al. (2023), has described the population structure, admixture history, and genomic diversity of Ethiopian cattle, the present study addresses a distinct question: whether closely related Ethiopian zebu populations inhabiting contrasting dry-hot and humid-hot environments show shared or environment-specific genomic signatures of heat-stress adaptation. By integrating within-population and cross-population selection scans, including iHS, Hp, XP-EHH, and XP-CLR, this study focuses on candidate loci and pathways that may have been shaped by divergent thermal and ecological pressures. Thus, the analysis extends previous population genomic work [11,25] by linking selection signatures to contrasting agro-ecological adaptation.

In this study, we reanalyzed publicly available whole-genome sequencing (WGS) data from 46 Ethiopian zebu cattle representing five populations inhabiting low-altitude regions (< 1500 m above sea level). Based on their ecological adaptation, these populations were grouped into Dry-Hot Ethiopian Zebu (DHETZ), comprising Afar, Ogaden, and Boran, adapted to dry-hot environments, and Humid-Hot Ethiopian Zebu (HHETZ), comprising Mursi and Goffa, adapted to humid-hot environments [11]. Given the low genetic differentiation but contrasting environmental pressures between DHETZ and HHETZ cattle, we hypothesized that these groups would share core thermotolerance-related selection signals while also displaying environment-specific adaptive signatures reflecting dry-hot versus humid-hot adaptation.

## Materials and methods

This study represents a secondary analysis of previously generated whole-genome sequencing (WGS) datasets. No new animal sampling, experimental procedures, or sequencing were performed as part of this study. WGS data were obtained from publicly available repositories, including the NCBI Sequence Read Archive and the China National Gene Bank Nucleotide Sequence Archive, as listed in S1 Table. Therefore, no additional ethical approval was required for the present secondary analysis.

### Agro-climatic characteristics of dry-hot and hot-humid agroecology

#### Dry-hot environment.

A dry-hot environment is defined by tropical arid conditions characterized by persistently high temperatures, low humidity, and minimal precipitation, typical of arid and semi-arid climates [26]. The Afar, Ogaden, and Borana plateaus, where Afar, Ogaden, and Boran cattle are predominantly raised, exemplify this agroecology. The annual mean temperature in these areas approximates 30 °C, with a seasonal variation from 11 °C in January to peaks of 45 ^°^C in June. Precipitation is scarce and highly variable, ranging from 2 mm in January to 56 mm in April.

#### Hot-humid environment.

Mursi and Goffa cattle are raised under humid-hot conditions. Goffa cattle are primarily located in the Goffa zone near Arbaminch, while Mursi cattle inhabit the South Omo zone. This geo-ecological region typically experiences relatively stable temperatures of 18 °C to 21 °C throughout the year, although extremes may fall to 10 °C to as high as 30 °C [26]. Rainfall is substantially higher than in dry-hot zones, with monthly precipitation ranging from 48 mm in January to 522 mm in October. Importantly, this humid-hot climate supports the proliferation of tsetse flies, which are closely linked to the transmission and prevalence of trypanosomes [27].

### Whole-genome sequencing data of Ethiopian cattle adapted to lowland environments

We reanalyzed publicly available WGS datasets from 46 Ethiopian cattle representing five populations: Afar (n = 9), Boran (n = 10), Goffa (n = 8), Mursi (n = 10), and Ogaden (n = 9). These cattle originate from low-altitude regions (< 1500 m above sea level) with distinct climatic pressures. Afar, Ogaden, and Boran cattle are adapted to dry-hot environments and collectively designated as the Dry-Hot Ethiopian Zebu (DHETZ) group. In contrast, Mursi and Goffa cattle thrive in humid-hot conditions, forming the Humid-Hot Ethiopian Zebu (HHETZ) group.

To investigate their genetic adaptations, we compared the Ethiopian cattle WGS data with those from non-Ethiopian cattle populations: 18 Sudanese zebu (SUZ: Butana n = 9 and Kenana n = 9), 27 Asian zebu (ASZ: Gir n = 10, Bhagnari n = 3, and *n* = 2 each from Achai, Cholistani, Dhanni, Gabrali, HisarHirayan, Sahiwal, and Tharparkar), 20 European taurine (EUT: Angus n = 10 and Holstein n = 10), and 20 African taurine (AFT: Muturu n = 10 and N’Dama n  = 10). All WGS datasets were retrieved from publicly available repositories, including the NCBI Sequence Read Archive (SRA) (https://www.ncbi.nlm.nih.gov/sra), and the China National Gene Bank (CNGB) Nucleotide Sequence Archive (CNSA) (https://db.cngb.org/cnsa/) (S1 Table).

### Read mapping and variant calling

Quality control of raw sequence reads was performed using FastQC v0.11.9 (https://github.com/s-andrews/FastQC/releases/tag/v0.11.9). Raw sequences with short reads (< 35 bp), low-quality bases (Phred score < 20), and sequence adapters were removed using Trimmomatic v0.38 [28]. The clean paired-end reads were then mapped to the *Bos taurus* reference genome ARS-UCD1.2 [29] using the Burrows-Wheeler Alignment (BWA-MEM) algorithm (BWA v.0.7.17) [30]. The mapped reads were sorted and indexed, and BAM files were produced using the SAMtools v1.8 [31]. Following alignment, PCR duplicate reads were identified and marked using the Picard MarkDuplicates tool in Picard v2.18.2 (https://broadinstitute.github.io/picard/). Subsequent processing included base quality score recalibration (BQSR) analysis, performed using Genome Analysis Toolkit (GATK) [32]. To enhance variant accuracy, the ARS1.2PlusY_BQSR_v3.vcf.gz dataset provided by the 1000 Bull Genomes Project Run 8 (http://www.1000bullgenomes.com/) was used for marking known sites in the bovine genome. The Variant Quality Score Recalibration (VQSR) was performed using the GATK VariantRecalibrator, utilizing known variant sources from dbSNP of the Bos taurus reference genome obtained from the Ensembl database (version 150). A sensitivity threshold between 99.9% and 100.0% was applied to assess the accuracy of variant mapping to the reference genome. A truth sensitivity threshold of 99.9% was used to filter the variants. After quality checks, variants from each sample were identified using the GATK HaplotypeCaller option, producing gVCF files. The gVCF from all samples was then combined to produce a joint genotype using the CombineGVCF option of GATK.

### Variant discovery and population genetic structure

SNPs were identified from the WGS data, and the summary statistics were computed using the vcf-stats of VCFtools v.0.1.15 [33]. Metrics recorded included the total number of biallelic SNPs, autosomal SNPs per sample, heterozygous-to-homozygous (Het/Hom) ratio, genomic inbreeding, and sequencing depth. These parameters were extracted using the BCFtools v.1.8 software [34]. Population genetic structure was assessed with the ADMIXITURE *v.1.3.0* [35]. Before admixture analysis, SNPs showing linkage disequilibrium (r^2^ > 0.5) within a 50 SNPs window and a 10 SNPs step were pruned from the datasets using the PLINK v1.9 [36]. The ancestral proportion was analyzed under various assumed backgrounds, ranging from K = 2 to K = 10. The optimal K value was determined by selecting the one that resulted in the lowest cross-validation error (S2 Table). Ancestry proportions across different K-values were visualized using the R package. To further investigate population differentiation, principal component analysis (PCA) was performed using the same PLINK on two datasets. The first dataset compares Ethiopian cattle groups (DHETZ and HHETZ) with non-Ethiopian populations (ASZ, SUZ, AFT, and EUT), while the second dataset contains only the Ethiopian cattle populations.

Population differentiation (F_ST_) analyses were performed using a balanced subset of 20 individuals per group where available. Because HHETZ included only 18 animals, all HHETZ individuals were retained, and 20 individuals were randomly selected from larger groups. To assess the effect of subsampling, F_ST_ estimates were compared with those obtained using all available individuals. The overall interpretation remained unchanged. Pairwise F_ST_ values were estimated using VCFtools v0.1.15 [33] within a 100 kb window and 50 kb step.

### Identification of selection signatures

To detect genomic regions under positive selection in Ethiopian cattle, we applied complementary within-population and cross-population selection-scan methods. Pooled heterozygosity (Hp) and integrated haplotype homozygosity (iHS) were used to detect selection signatures within DHETZ and HHETZ. Cross-population extended-haplotype homozygosity (XP-EHH) and composite likelihood ratio (XP-CLR) were used to compare DHETZ and HHETZ with Asian zebu and with each other [37]. For iHS and XP-EHH analysis, genotypes were phased and imputed using the BEAGLE v5.1 [38]. Because Ethiopian zebu-specific reference panels are limited, we acknowledge that phasing and imputation uncertainty may have influenced haplotype-based statistics in our studies. To minimize overinterpretation, iHS results were interpreted together with other selection scans, and high-confidence candidate regions were defined as those supported by at least three of the four methods.

Hp was calculated from individual-level WGS genotype data by estimating heterozygosity within sliding genomic windows and identifying windows showing reduced heterozygosity relative to the genome-wide distribution. Although Hp was originally developed for pooled sequencing data, it has also been applied to window-based summaries of individual-level resequencing data to identify regions of reduced genetic diversity consistent with candidate selective sweeps. The regions were studied in a 100 kb window size and 50 kb window step size using the equation Hp = 2∑*nMAJ* ∑*nMIN*/(∑*nMAJ* + ∑*nMIN*)^2^ [37], where ∑*nMAJ* and ∑*nMIN* are the sum of major and minor alleles in a window, respectively. For XP-EHH and XP-CLR analyses, we used a 100 kb window size and a 50 kb step size to identify recent positive natural selection between two populations. This window size was selected to balance genomic resolution with robustness to local variation in SNP density and to facilitate comparison across selection statistics. XP-EHH*,* used to identify recent positive natural selection in the genome [39], was calculated using the rehh pakage v2.0 in R v4.2 [40]. The XP-CLR, used to detect recent selective sweeps by comparing allele frequency differences between two populations, was calculated using the xpclr v1.1.2 [41]. The significance of candidate regions and the threshold level of each selection scan method were determined at the top 0.5% windows.

### Functional annotation and characterization of candidate genes

Genomic regions identified by selection-scan methods were annotated using the Ensembl Biomart based on the ARS-UCD1.2 bovine reference genome. Protein-coding genes located within the significant candidate regions were extracted and used for functional enrichment analysis. Gene Ontology (GO) biological process and molecular function, and Kyoto Encyclopedia of Genes and Genomes (KEGG) pathway enrichment analyses were performed using the Database for Annotation, Visualization, and Integrated Discovery (DAVID) bioinformatics tool (https://davidbioinformatics.nih.gov/home.jsp) [42]. The *Bos taurus* genome background was used to run functional annotation analysis of the protein-coding genes. Genes that were clustered in GO terms and KEGG pathways with fold enrichment > 1.0 and Fisher’s exact-test P < 0.05 were considered enriched.

## Results

### Variant discovery and population structure

Following stringent quality control of WGS data, we identified 34.2, 33.7, 29.4, 32.2, 16.1, and 13.8 million biallelic autosomal SNPs in HHETZ, DHETZ, SUZ, ASZ, AFT, and EUT cattle, respectively (Table 1; S1 Table). Of these, 93.4%, 91.7%, 93.0%, 87.5%, 93.5%, and 95.1% are known SNPs, mapped to the ARS-UCD1.2 taurine cattle reference genome. The average number of SNPs per sample ranges from 13.8 million in ASZ to 5.7 million in AFT and EUT cattle, with taurine cattle consistently exhibiting fewer SNPs compared to zebu populations.

**Table 1:** Summary of SNP statistics, including total SNP count, average SNPs per sample, heterozygosity ratio, and genomic inbreeding across the studied cattle populations.

Group	HHETZ	DHETZ	SUZ	ASZ	AFT	EUT
Total biallelic autosomal SNPs	34,207,856	33,760,783	29,460,635	32,284,391	16,147,621	13,837,429
Average SNPs per sample	12,265,764	12,140,945	11,872,944	13,886,220	5,764,851	5,723,518
Private SNPs	79,191	63,691	217,727	139,629	143,273	96,965
Het/non-ref-hom ratio	1.71	1.43	1.40	2.30	0.80	1.60
Genomic inbreeding	0.091	0.197	0.166	0.383	0.681	0.597

DHETZ = Ethiopian zebu living in a dry-hot environment, HHETZ = Ethiopian zebu living in a humid-hot environment, AFT = African taurine, ASZ = Asian zebu, EUT = European taurine, SUZ = Sudanese zebu, Het = heterozygous, non-ref-hom = non-reference homozygous.

Ethiopian zebu groups (DHETZ and HHETZ) display comparable SNP counts, despite inhabiting contrasting agro-ecologies (dry-hot vs. humid-hot). In contrast, ASZ cattle display the highest per-sample average SNP count . Both DHETZ and HHETZ harbor fewer private SNPs relative to the reference genome, yet they exhibit higher total SNP counts and heterozygosity-to-homozygosity ratios, consistent with increasing genetic diversity (Table 1).

The heterozygous-to-homozygous ratio varies significantly across populations, with values of 1.71 in HHETZ and 1.43 in DHETZ. These are lower than those in ASZ cattle (2.3) but higher than in AFT cattle (0.8). Ethiopian cattle exhibit lower levels of genomic inbreeding, with HHETZ showing the highest heterozygosity and lowest inbreeding. While SUZ cattle also show elevated heterozygosity, which may reflect recent admixture. DHETZ and SUZ cattle showed comparable heterozygosity and inbreeding.

Population differentiation analysis reveals the lowest genetic differentiation between HHETZ and DHETZ (F_ST_ = 0.0063). Both groups also cluster closely with SUZ cattle, with F_ST_ = 0.0242 and 0.0231, respectively, reflecting their geographic proximity and shared ancestry. Among Ethiopian groups, DHETZ shows the closest affinity to SUZ cattle, consistent with their common history and adaptation to dry-hot environments (Table 2). A broader comparison showed that African zebu (HHETZ, DHETZ, and SUZ cattle) share a more similar genetic structure relative to ASZ. Across all pairwise comparisons, the highest genetic differentiation was observed between taurine cattle (AFT and EUT) and ASZ (Table 2).

**Table 2:** Population differentiation among the cattle groups in the dataset.

	HHETZ	DHETZ	SUZ	ASZ	AFT
**DHETZ**	0.0063	0			
**SUZ**	0.0242	0.0231	0		
**ASZ**	0.0644	0.0572	0.0658	0	
**AFT**	0.2370	0.2564	0.2500	0.3458	0
**EUT**	0.2770	0.2933	0.2889	0.3681	0.1946

DHETZ = Ethiopian zebu living in a dry-hot environment, HHETZ = Ethiopian zebu living in a hot-humid environment, AFT = African taurine, ASZ = Asian zebu, EUT = European taurine, SUZ = Sudanese zebu.

**Fig 1:**
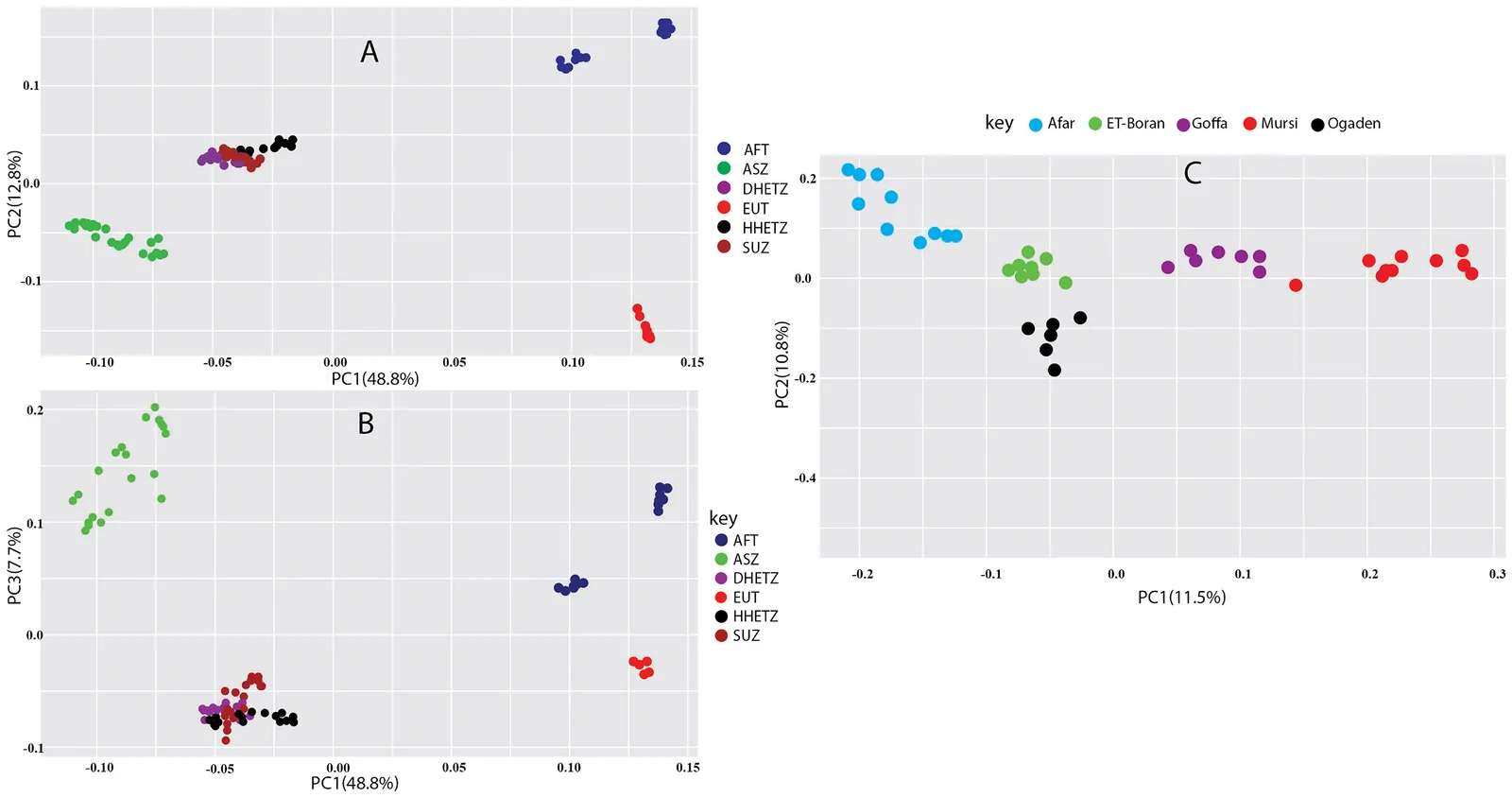
Principal component analysis result. (A) PC1 and PC2 of all populations in the dataset. (B) PC1 and PC3. (C) PC1 and PC2 of Ethiopian cattle populations adapted to a dry-hot environment (Afar, Boran, and Ogaden), and a humid-hot environment (Goffa and Mursi). Populations adapted to a dry-hot environment (DHETZ), humid-hot environment (HHETZ), Sudanese zebu (SUZ), African taurine (AFT), Asian zebu (ASZ), and European taurine (EUT) cattle.

Two PCA analyses were performed. The first included a dataset containing all cattle populations and showed that PC1 and PC2 explained 48.8% and 12.8% of the total variance, respectively. PC1 separated the zebu populations (DHETZ, HHETZ, SUZ, and ASZ cattle) from taurine cattle (AFT and EUT) while PC2 distinguished African cattle (DHETZ, HHETZ, SUZ, and AFT) from non-African populations (EUT and ASZ) (Fig 1A). In this group, PC1 and PC3 (7.7%) separated ASZ and AFT from African zebu (DHETZ, HHETZ, and SUZ cattle) and EUT cattle (Fig 1B). Ethiopian zebu cattle (DHETZ and HHETZ) clustered closely with SUZ cattle, reflecting their shared zebu ancestry and geographical proximity.

The second PCA was restricted to a dataset containing only Ethiopian cattle populations. In this analysis, PC1 and PC2 explained 11.5% and 10.8% of the variance, respectively. Within Ethiopian cattle, PC1 clearly differentiated DHETZ (Afar, Ogaden, and Boran) from the HHETZ (Mursi and Goffa), consistent with their contrasting agro-ecological backgrounds. However, PC2 did not reveal further sub-structuring among Ethiopian groups (Fig 1C).

**Fig 2:**
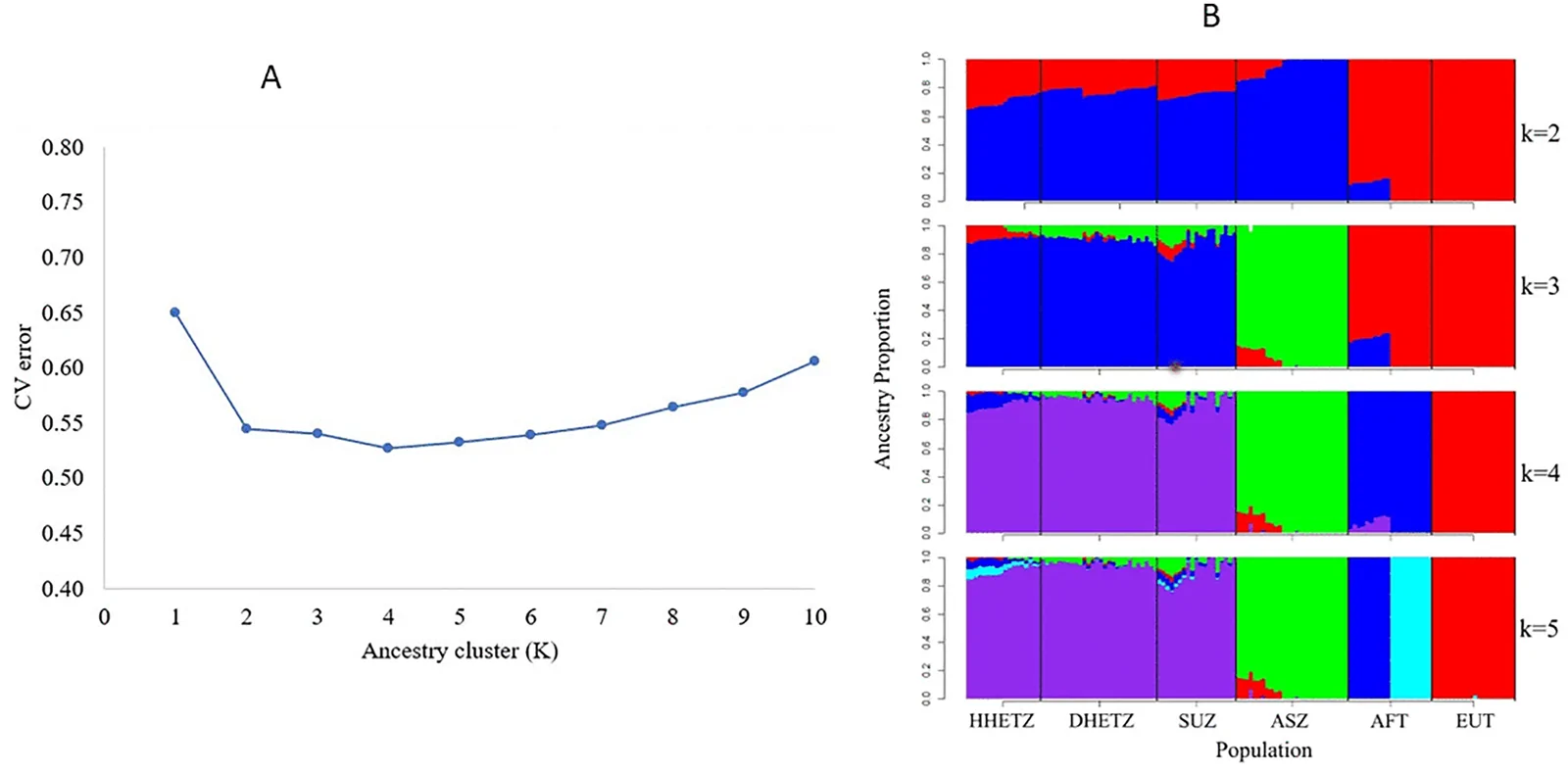
Population genetic structure. A) Validation error coefficient all cattle group admixture analysis, B) admixture plot of whole population dataset containing the Ethiopian zebu cattle in a dry-hot environment (DHETZ) and humid-hot environment (HHETZ), Sudanese zebu (SUZ), African taurine (AFT), Asian zebu (ASZ), and European taurine (EUT) cattle.

Genetic admixture analysis was conducted using autosomal biallelic SNPs after removing linked SNPs (r^2^ > 0.5). The optimal number of ancestral clusters is K = 4 (Fig 2A, S2 Table). At this level, Ethiopian zebu (ETZ) includes two indicine-specific ancestries, one African (93.3%) and a minor one shared with Asian zebu (2.8%), and two taurine-specific ancestries, an African taurine (3.4%) and a minor one (0.5%) shared with European taurine. At K = 3, HHETZ shows a higher proportion of taurine ancestry than DHETZ (Fig 2B), whereas DHETZ shares more zebu ancestry with ASZ. Additionally, a comparable ASZ component is observed in SUZ cattle compared to DHETZ. At K = 5, the African taurine background, exemplified by the N’Dama, is detected at a higher proportion in HHTZ than in DHETZ cattle.

### Selection signature within the Ethiopian cattle populations

#### Dry-hot environment (DHETZ).

**Fig 3:**
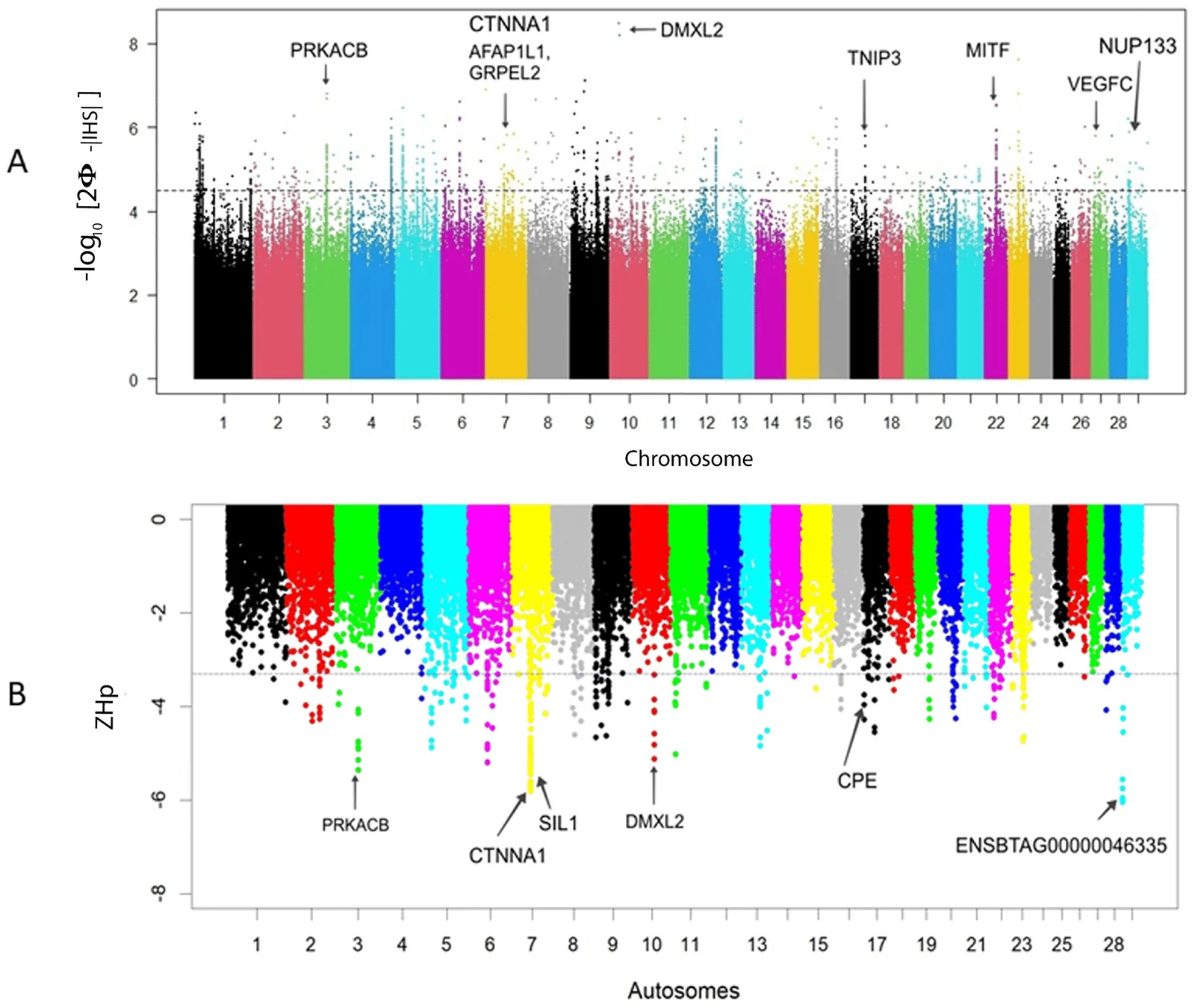
Manhattan plots of genome regions detected by (A) iHS and (B) Hp in DHETZ. Candidate genes annotated on each plot are genes identified by at least three of the four selection scan methods (iHS, HP, XP-EHH, and XP-CLR).

To identify genomic regions under selection in Ethiopian cattle inhabiting dry-hot environments (DHETZ), we employed the iHS and Hp analyses. A total of 298 non-overlapping genome regions were identified by the iHS, at a threshold of P < 3.96 × 10^-5^ (Fig 3A), encompassing 502 protein-coding genes annotated against the Ensembl ARS-UCD1.2 taurine reference genome (S3 Table). The Hp scan identified 113 non-overlapping candidate genome regions under selection at a threshold value ZHp < −3.3 (Fig 3B), overlapping with 183 protein-coding genes (S4 Table). Together, the iHS and Hp detected 33 candidate genes in common, spanning 2.7 Mb across BTA 2, 3, 5, 6, 7, 10, 11, 13, 18, 20, 28, and 29.

#### Humid-hot environment (HHETZ).

**Fig 4:**
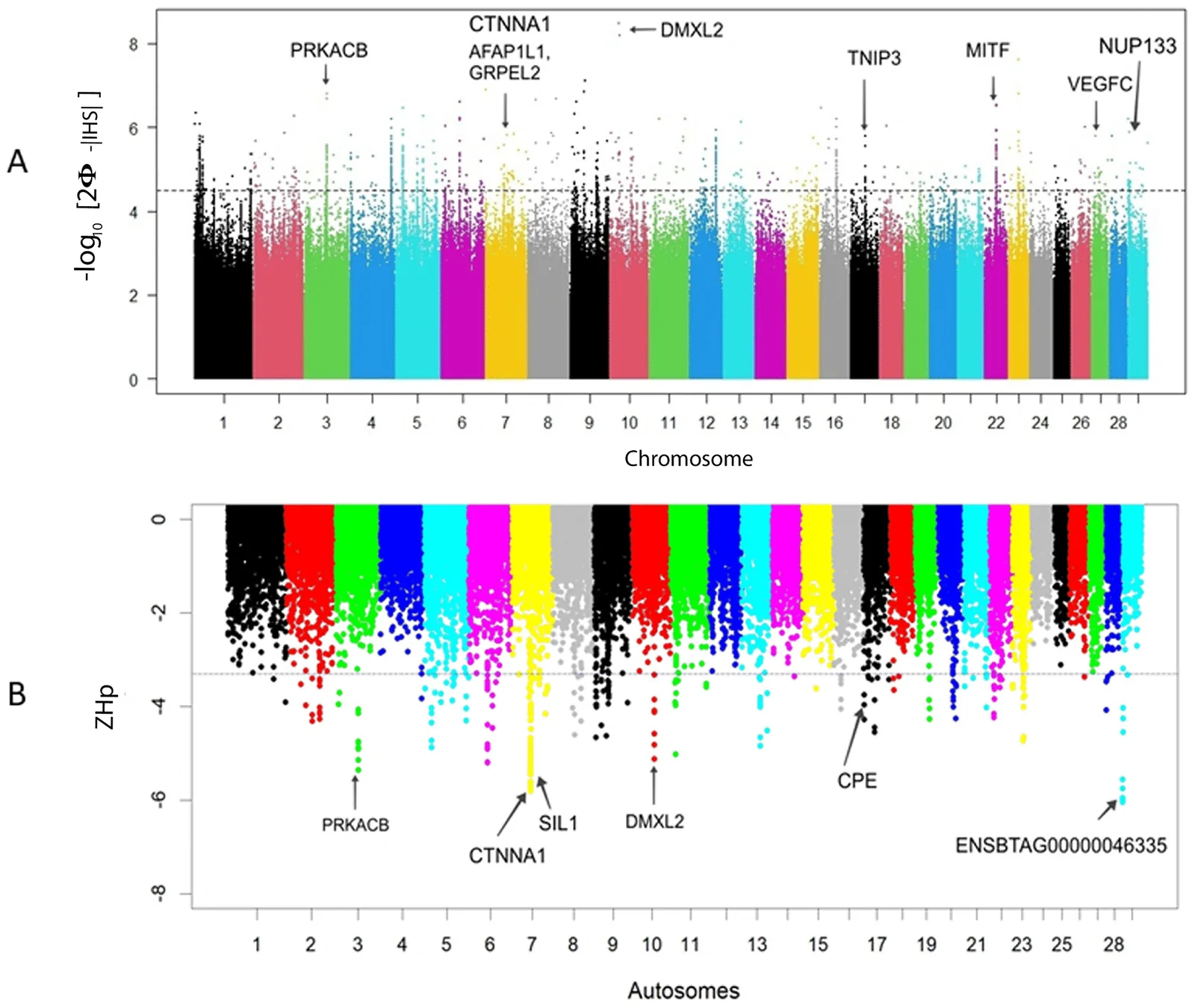
Manhattan plots of candidate genome regions detected by (A) iHS and (B) Hp selection scan methods in HHETZ cattle. Candidate genes annotated on each plot are genes identified by at least three of the four methods (iHS, Hp, XP-EHH, and XP-CLR). To evaluate consistency across selection-scan methods, we calculated the overlap between candidate regions and genes detected by within-population and cross-population approaches. Only loci supported by at least three of the four methods were prioritized as high-confidence candidates.

Applying the same iHS and Hp methods to cattle adapted to hot-humid environments (HHETZ) revealed 244 and 138 non-overlapping genome regions, respectively, at a threshold of iHS = 5.2 (P = 6.22 × 10^-6^) and ZHp *<* −3.2 (Figs 4A and 4B). These regions overlap with 339 (iHS) and 183 (Hp) candidate genes (S5 and S6 Tables). A total of twenty-six candidate genes were identified using both methods. These genes are distributed across BTA 5, 6, 9, 10, 12, 13, 15, and 19. They cover 2.5 Mb of the genome.

### Comparative genomic signatures of selection among DHETZ, HHETZ, and ASZ populations

#### DHETZ and HHETZ.

To identify divergent selection signals between DHETZ and HHETZ populations, we employed XP-EHH and XP-CLR) analyses. The XP-EHH, which measures haplotype decay between populations, detected 163 non-overlapping genomic regions (100 kb windows), spanning 27.15 Mb, and harboring 329 protein-coding genes. In parallel, the XP-CLR identified 227 non-overlapping genomic regions totaling 23.9 Mb and overlapping with 332 protein-coding genes (S7 and S8 Tables). A total of 10 genomic regions containing 20 candidate genes are shared between XP-EHH and XP-CLR results. They are localized on BTA 1, 2, 6, 7, 8, 17, 23, and 28 (Table 5). These overlapping signals represent strong candidates for loci underlying adaptive differentiation between cattle inhabiting dry-hot *versus* hot-humid environments.

#### DHETZ and ASZ.

**Fig 5:**
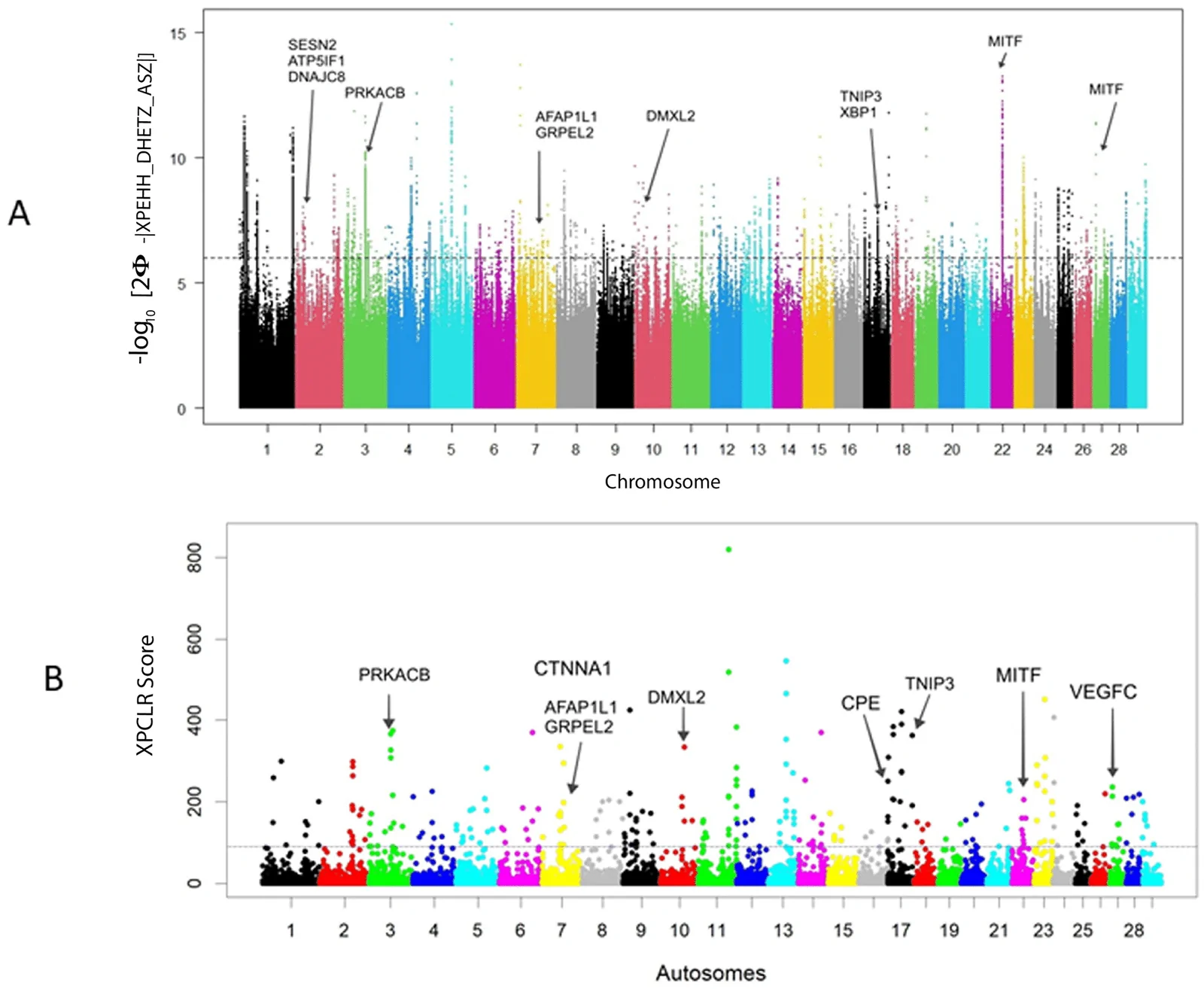
Manhattan plots of genome regions detected between DHETZ and ASZ cattle. (A) XP-EHH and (B) XP-CLR. Candidate genes annotated on each plot are genes identified by at least three of the four methods (iHS, Hp, XP-EHH*,* and XP-CLR).

Comparative genomic scans between DHETZ and ASZ cattle using XP-EHH at a threshold value P < 1.0 × 10^-6^ and XP-CLR (score ≥ 90) identified 270 and 207 non-overlapping genome regions, respectively (Figs 5A and 5B). These genome regions were associated with 614 (XP-EHH) and 271 (XP-CLR) protein-coding genes (S9 and S10 Tables). Across both methods, a total of 56 protein-coding genes were detected. They are located on BTA 2, 3, 4, 5, 6, 7, 10, 11, 12, 16, 17, 22, 23, 27, 28, and 29.

#### HHETZ and ASZ.

**Fig 6:**
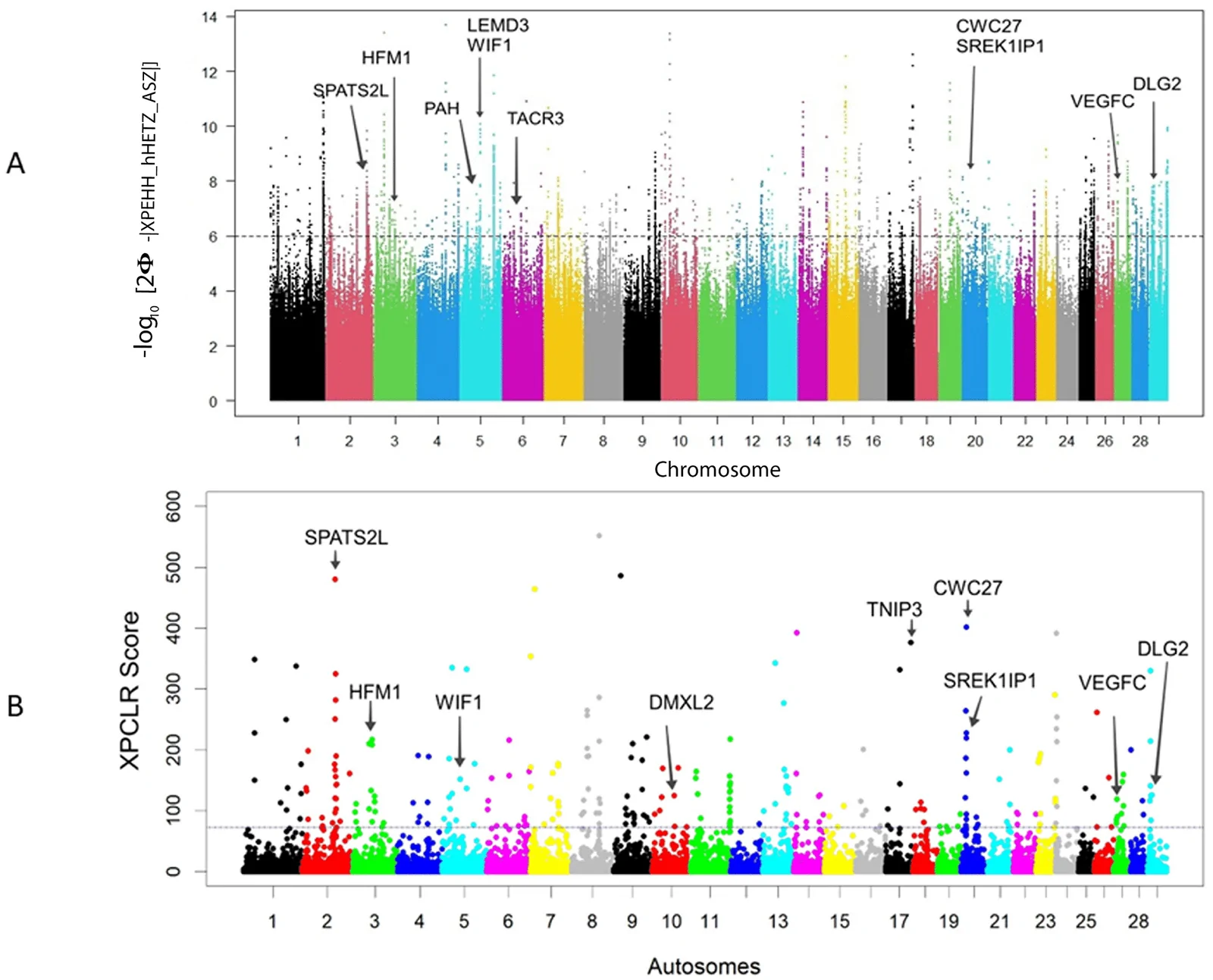
Manhattan plots of candidate genome regions detected between HHETZ and ASZ cattle. (A) XP-EHH, and (B) XP-CLR. Candidate genes annotated on each plot are genes identified by at least three of the four methods (iHS, Hp, XP-EHH, and XP-CLR).

Using the same cross-population tests, 257 and 215 non-overlapping regions were identified between HHETZ and ASZ cattle, within the top 0.5% of 100 kb windows, corresponding to XP-EHH ≥ 6.0 (P < 1.0 × 10^-6^) and XP-CLR score ≥ 75 (Figs 6A and 6B). These regions encompassed 644 (*XP-EHH*) and 297 (XP-CLR) protein-coding genes (S11 and S12 Tables). Across both methods, 24 candidate genes are shared. They are distributed across BTA 2, 3, 5, 8, 14, 20, 27, and 29.

### Overlap selection scans candidate genes and functional annotation

#### DHETZ.

To assess consistency among selection-scan methods, we compared the overlap of candidate regions and genes detected by the iHS, Hp, XP-EHH, and XP-CLR. Candidate regions supported by at least three of the four methods were considered as high-confidence regions. Integration of signals from all four selection scans identified 19 high-confidence candidate regions in DHETZ. These regions, spanning 2.32 Mb, were distributed across BTA 2, 3, 7, 10, 16, 17, 20, 22, 23, 27, 28, and 29. They include 38 protein-coding genes, e.g., *SESN2*, *ATP5IF1*, *DNAJC8*, *SAMD13*, *PRKACB*, *TTLL7*, *CTNNA1*, *SIL1*, *ABLIM3*, *AFAP1L1*, *DMXL2*, *GLDN*, *USH2A*, *CPE*, *XBP1*, *TNIP3*, *ADAMTS12*, *MITF*, *VEGFC*, *NUP133*, *ABCB10*, *URB2,* along with several olfactory receptor genes (Table 3). These candidate genes are functionally linked to key traits in adaptation to heat stress, including oxidative stress response, protein folding, heat shock response, melanogenesis, coat pigmentation, reproduction, and angiogenesis.

**Table 3:** List of candidate regions and associated genes identified in DHETZ cattle by at least three genomic scan methods.

BTA	Region Start (Mb)	Region End (Mb)	HP	iHS	XP-EHH	XP-CLR	Gene name	Phenotype	Reference
2	125.10	125.4	N.D.	4.6	7.2	106.7	*SESN2, ATP5IF1, DNAJC8*	Oxidative stress response, unfolded protein, and heat shock protein response.	[43,44]
3	59.75	60.20	−3.9	6.9	11.6	135.8	*SAMD13*	Heat shock protein	[43]
3	59.95	60.05	−5.4	6.9	11.6	367.5	*PRKACB*	Melanogenesis	[45]
3	60.05	60.15	−4.3	6.9	11.6	N.D.	*TTLL7*	Protein modification /polyglutamylation in the central nervous system	[46]
7	49.95	50.10	−5.2	6.4	6.3	335.7	*CTNNA1*	Skeletal muscle development	[47]
7	50.20	50.30	−5.0	N.D.	6.3	164.1	*SIL1*	Unfold protein binding and cochaperone protein ER HSPAS	
7	60.50	60.70	N.D.	5.2	6.5	294.6	*ABLIM3,*	Actin-binding, striated muscle activator	[48]
			N.D.	5.2	6.5	198.4	*AFAP1L1, GRPEL2*	Adaptor protein activates mtHSP70 and regulates oxidative stress.	[49,50]
10	58.85	59.05	−4.4	6.4	6.8	211.8	*DMXL2*	Nerve development that regulates fertility through GnRH secretion	[51,52]
			−4.1	6.4	N.D.	188.3	*GLDN*	Nervous system development	[53]
16	19.5	19.6	−3.9	N.D.	6.7	113.4	*USH2A*	Vision and hearing	[54]
17	0.57	0.72	−3.4	N.D.	7.8	251	*CPE*	Cell proliferation and inhibition of ischemia/hypoxia-induced apoptosis	[55]
17	67.95	68.25	N.D.	4.7	6.6	190	*XBP1*	Unfolded proteins in the endoplasmic reticulum, the MHC-class II gene enhancer	[56–58]
			N.D.	4.7	6.6	363.9	*TNIP3*	Regulate inflammation, cell death, and Cardiac hypertrophy.	[59]
20	39.90	40.20	−3.7	4.9	N.D.	92.4	*ADAMTS12*	Upregulating VGEF promotes angiogenesis,	[60]
22	31.60	31.7	N.D.	5.3	13.2	205.2	*MITF*	Coat color pigmentation	[61]
27	80.50	81.50	N.D.	5.5	6.7	236.2	*VEGFC*	Angiogenesis	[62]
28	1.45	1.50	N.D.	5.0	6.8	209.1	*NUP133, ABCB10*	regulate mitochondrial function and oxidative stress, reactive oxygen species	[63,64]
	1.45	1.70	−3.5	5.0	6.8	N.D.	*URB2*	hematopoietic stem cell development through the regulation of the p53/TP53 pathway	[65]
29	0.20	0.30	−5.5	10.8	N.D.	111.8	ENSBTAG00000054019		
			−5.5	10.8	N.D.	201.7	ENSBTAG00000046335		

BTA, Bos taurus autosome; Region Start and Region End are presented in megabase (Mb) based on the ARS-UCD1.2 bovine reference genome,, Hp, pooled heterozygosity; iHS, integrated haplotype score; XP-EHH, cross-population extended haplotype homozygosity; XP-CLR, cross-population composite likelihood ratio. N.D., not detected by the corresponding method.

Functional annotation clustering revealed enrichment in the GO biological processes such as cellular response to unfolded proteins (GO:0034620), transmembrane receptor protein kinase activity (GO:0019199), response to biotic stimulus (GO:0071216), signal transduction regulation (GO:0009966), and the cellular response to lipopolysaccharide (GO:0071222). Enriched molecular functions included enzyme binding (GO:0019899) and NF-kappa-B binding (GO:0051059). The KEGG pathways analysis indicated enrichment in the thyroid hormone signaling pathway (bta04919) and neutrophil extracellular trap formation (bta04613) (S13 Table).

Among the functionally enriched genes, *XBP1*, *ABCB10*, and *TMBIM4* are linked to unfolded protein response and cellular homeostasis during environmental stress. Additional genes such as *XBP1*, *TNIP3*, *PLCG2*, and *IRAK3*, are involved in lipopolysaccharide response. These genes may contribute to the immune response under stress. Collectively, these genes highlight molecular pathways that may be involved in resilience to heat stress in dry-hot environments.

#### HHETZ.

The overlap of candidate genes detected by HP and iHS within the HHETZ, and by XP-EHH and XP-CLR between the HHETZ and ASZ cattle, is summarised in Table 4. Among genomic regions under positive selection, 13 candidate regions were identified by at least three of the four methods. These regions, spanning 2.6 Mb across BTA2, 3, 5, 6, 10, 12, 15, 20, 27, and 29, overlap with 14 protein-coding genes, including *SPATS2L*, *HFM1*, *LEMD3*, *WIF1*, *PAH*, *TACR3*, *DMXL2*, *FRY*, *B3GLCT*, *SOX6*, *CWC27*, *SREK1IP1*, *VEGFC*, and *DLG2* (Table 4). Among these, *WIF1* was the most consistently supported signal in HHETZ, as it was detected by all four selection-scan methods. It is located on BTA5, spanning 48.65 to 48.75 Mb, and is functionally associated with anti-angiogenesis. This makes *WIF1* the strongest HHETZ-specific candidate gene in the present analysis and suggests a potential role for Wnt-related vascular regulation under hot-humid environmental conditions.

**Table 4:** Candidate gene regions and associated genes identified in HHETZ cattle by at least three genomic scan methods.

BTA	Region Start (Mb)	Region End (Mb)	HP	iHS	XP-EHH	XP-CLR	Gene name	Phenotype	Reference
2	88.80	88.90	−3.6	N.D.	7.1	480.2	*SPATS2L*	Type I interferon, responsible for lupus	[66]
3	51.85	52.00	−4.3	N.D.	6.9	217	*HFM1*	Gamete formation, Reproduction	[67]
5	48.45	49.00	−3.3	6.8	7.5	N.D.	*LEMD3*	Regulate cytokine family, like TGF-β, BMPs	[68]
5	48.65	48.75	−3.7	6.8	7.5	151.9	*WIF1*	Anti-angiogenesis	[69]
5	66.60	66.70	−3.7	7.1	N.D.	136.5	*PAH*	Activate tyrosine to produce melanin	[70,71]
6	21.40	21.65	−3.4	8.8	6.7	N.D.	*TACR3*	Gonad development and reproduction	[72]
10	58.85	58.95	−3.3	5.2	N.D.	124.9	*DMXL2*	Fertility	[51,52]
12	28.90	29.20	−3.3	6.8	6.4	N.D.	*FRY*	Mammary gland development	[73]
12	29.60	29.75	−3.9	5.5	6.8	N.D.	*B3GLCT*	Environmental adaptation	[25,74]
15	35.70	35.85	−3.4	6.5	N.D.	73.2	*SOX6*	Growth and muscle development	[75]
20	14.40	14.70	−3.8	N.D.	7.2	401.9	*CWC27*	Vision, retinal development	[76]
			−3.8	N.D.	7.2	219	*SREK1IP1*	Activate Vitamin D, related genes	[77]
27	7.95	8.15	N.D.	5.3	7.1	85.2	*VEGFC*	Angiogenesis	[62]
29	12.05	12.20	N.D.	5.6	7.8	125.56	*DLG2*	Puberty development	[78]

BTA, Bos taurus autosome; Region Start and Region End are presented in megabases (Mb) based on the ARS-UCD1.2 bovine reference genome. Hp, pooled heterozygosity; iHS, integrated haplotype score; XP-EHH, cross-population extended haplotype homozygosity; XP-CLR, cross-population composite likelihood ratio. N.D., not detected by the corresponding method.

GO and KEGG pathway enrichment analyses of candidate genes detected in HHETZ (S14 Table) highlighted biological processes such as immune response-activating cell surface signaling (GO:0002429), cell surface receptor signaling (GO:0007166), homeostatic process (GO:0042592), and immune response-activating signaling (GO:0002757). Significant molecular functions included glycosyltransferase activity (GO:0016757), and gated channel activity (GO:0022836).

KEGG pathway analysis further revealed enrichment in the thyroid hormone signaling pathway (bta04919), MAPK signaling pathway (bta04010), and African trypanosomiasis (bta05143). Notably, candidate genes *MYD88*, *ENSBTAG00000048268*, ENSBTAG00000050088, and *ENSBTAG00000053508* were grouped within the trypanosomiasis pathway. MYD88 (BTA21) and associated genes on BTA22 are essential for the innate immune system and immunoglobulin-mediated immune response, potentially enhancing resistance to trypanosome infections in HHETZ.

### Common candidate genes detected between DHETZ and HHETZ

A total of nine common genomic regions were identified between DHETZ and HHETZ using the XP-EHH and XP-CLR (Table 5). These regions contain 20 candidate genes (*MBNL1, DOCK10, RHBDD1, COL4A4, APBB2, NR3C1, RAD50, SMARCB1, DERL3, SLC2A11, MIF, GSTT4, GSTT1, GSTT2, DDT, CABIN1, RAB44*, ENSBTAG00000052065, ENSBTAG00000053662, and *RET*). These loci are distributed across BTA 1, 2, 6, 7, 8, 17, 23, and 28, spanning approximately 1.8 Mb.

**Table 5:** Candidate gene regions and associated genes identified in DHETZ and HHETZ cattle.

BTA	Region Start (Mb)	Region End (Mb)	XP-EHH	XP-CLR	Gene name	Phenotype function	Reference
1	115.5	116.0	16.2	75.6	*MBNL1*	RNA splicing and regulation play essential roles in muscle development	[79]
2	112.70	112.95	8.9	36.9	*DOCK10*	Associated with immune response, lipid metabolism, meat color, and intramuscular fat	[80]
2	115.05	115.20	9.8	52.9	*RHBDD1*	Lipid metabolism, growth, and development,	[81]
				52.9	*COL4A4*	Structural integrity in tissues, immune function, and parasite resistance	[82]
6	59.95	60.10	9.1	44.3	*APBB2*	Involved in fatty acid biosynthesis and lipid metabolism related to carcass quality.	[83]
7	54.45	54.60	9.7	24.8	*NR3C1*	Plays an important role in stress and inflammation response.	[84]
7	21.80	21.95	8.3	38.6	*RAD50*	DNA double-strand break repair contributes to DNA damage response and response to UV damage	[85]
8	12.45	12.55	9.8	15.9	ENSBTAG00000052065	Gene Associated with Endoparasite Infection	[86]
17	71.20	71.35	9.1	18.8	*SMARCB1*	ATP-dependent chromatin-remodeling complex that regulates gene expression, plays an important role in cell proliferation and differentiation.	[87]
					ENSBTAG00 000053662	Glutathione metabolic process, responsible for detoxification and antioxidant factor	[88]
					*DERL3*	Heat stress response, Endoplasmic Reticulum Stress	[89]
					*SLC2A11*	Glucose transport across cell membranes, linked to pigment differentiation,	[90]
					*MIF*	Innate immune system	[91]
					*GSTT4, GSTT2, GSTT1*	Detoxification and stress responses. Xenobiotic resistance, Cell detoxification, responses to oxidative stress, muscle development, and growth	[88,92]
					*DDT*	Involved in the Melanin Synthesis Pathway, melanogenesis	[91]
					*CABIN1*	Neuron development plays a significant role in calcium signaling in calcium-dependent cellular processes, and Muscle development	[93]
23	10.60	10.75	8.9	26.7	*RAB44*	Immunity response and melanosome transport in melanocytes	[94]
28	13.35	13.50	9.0	20.2	*RET*	Neuron development and signal transduction activate heat shock transcription factor (HSTF1) in response to heat stress.	[95]

BTA, Bos taurus autosome; Region Start and Region End are presented in megabase (Mb) based on the ARS-UCD1.2 bovine reference genome. Hp, pooled heterozygosity; iHS, integrated haplotype score; XP-EHH, cross-population extended haplotype homozygosity; XP-CLR, cross-population composite likelihood ratio. N.D., not detected by the corresponding method.

The functional annotation of these genes reveals significant enrichment in biological processes linked to signal transduction regulation (GO:0009966), reproduction (GO:0022414), and molecular adaptor activity (GO:0060090). KEGG pathways included the PI3K-Akt signaling pathway (bta04151) and melanoma (bta05218), both relevant to stress response and cellular resilience. Several genes (*RAD50*, *NR3C1*, *RHBDD1*, and *KIT*) were implicated in reproductive processes, including meiosis, gametogenesis, and reproductive organ function (S15 Table), underscoring their role in adaptive traits across environmental and developmental contexts.

## Discussion

The ability of African zebu cattle to thrive in extreme thermal environments is driven by genetic adaptations that enhance thermoregulation, oxidative stress tolerance, metabolic efficiency, and immune competence. Deciphering the genetic bases of these traits is critical for sustaining livestock productivity in tropical and arid regions, particularly under the increasing threat of climate change-induced heat stress [26]. In this study, we investigated the genomic architecture of Ethiopian zebu cattle from dry-hot (DHETZ) and humid-hot (HHETZ) environments, comparing their diversity and selection patterns with Asian zebu (ASZ), Sudanese zebu (SUZ), and taurine cattle.

The present findings build on previous work by Terefe et al. (2022, 2023) [11,25], which characterized population structure, admixture history, genomic diversity, and high altitude adaptation in Ethiopian cattle. While these studies established the broader genetic relationships among Ethiopian cattle populations, the present analysis focused specifically on signatures of selection associated with contrasting hot-arid and hot-humid environments. The low genetic differentiation between DHETZ and HHETZ supports their close shared ancestry; however, the detection of both shared and environment-specific selection signals suggests that ecological pressures may have acted on partially overlapping adaptive pathways. Thus, the current study extends previous population-genomic analyses by linking selection signatures to contrasting agro-ecological adaptation rather than describing population structure alone. The population structure and admixture analyses reveal that the Ethiopian zebu cattle (DHETZ and HHETZ) share a strong African-specific zebu ancestry and cluster closely with Sudanese zebu. However, they diverge significantly from ASZ and taurine breeds. Principal component and admixture analyses highlight minimal differentiation between DHETZ and HHETZ, suggesting a common ancestral origin. Environment-specific selection pressures or population-specific demographic events might have shaped the small divergence. DHETZ clustered more closely with SUZ cattle, consistent with their geographic proximity and shared adaptation to arid environments [11]. Overall, our findings emphasize the evolutionary plasticity of the zebu genome in response to distinct environmental challenges across diverse ecological niches [23,96].

Heat stress can disrupt protein folding, cause cellular damage, impair metabolism, and impact reproductive physiology. To mitigate these effects, molecular chaperones and stress regulators play essential roles in maintaining protein stability and cellular homeostasis [57,97]. In DHETZ, selection scans identified several key candidate genes, including *DNAJC8*, *SAMD13*, *XBP1*, and *ABCB10*, which are critical for protein refolding, mitochondrial protection, and stress-induced endoplasmic reticulum (ER) homeostasis [43,56]. Additionally, candidates include *ABLIM3* and *AFAP1L1* (BTA7:60.6–60.7 Mb), which contribute to muscle fiber integrity, actin cytoskeleton organization, and endothelial proliferation—mechanisms that support vascular remodeling and thermoregulation [48,98]. *ABLIM3*, a member of the actin-binding LIM family of proteins, functions as a cytoskeletal adaptor linking actin filaments to signaling pathways, thereby influencing cell shape, movement, and stress responses [99]. *AFAP1L1*, which encodes an actin filament-associated protein, is involved in muscle development and fat deposition, potentially influencing carcass yield and quality.

The *XBP1* transcription factor is central to the unfolded protein response, regulating genes involved in ER stress recovery, protein folding, and ER-associated degradation. Beyond stress tolerance, *XBP1* also contributes to lipid and glucose metabolism, immune responses, and cellular development [100]. Similarly, *GRPEL2*, a mitochondrial chaperone linked to the *ABLIM3* and *AFAP1L1* region, regulates HSP70 activity and ATP-dependent protein folding. By reducing oxidative stress and preserving mitochondrial function, *GRPEL2* enhances resilience to fluctuating temperatures [50,101].

Heat stress also elevates reactive oxygen species, leading to oxidative damage and metabolic imbalance [102]. The *SESN2* gene, located on BTA2 and selected in DHETZ, functions as an antioxidant regulator that maintains redox balance, prevents oxidative stress-induced apoptosis, and safeguards mitochondrial function [103]. These results highlight *SESN2* as a promising candidate locus for heat-stress resilience.

In HHETZ, strong selection signals were associated with metabolic adaptation, reproduction, immune defense, and resistance to vector-borne diseases, particularly trypanosomiasis. Metabolic plasticity is a crucial adaptive mechanism for efficient energy utilization under heat stress [5]. Genes such as *PRKACA* and *PRKACB* regulate lipolysis in adipocytes, mobilizing fat reserves to sustain energy production during thermal challenges [104,105].

Immune-related genes under selection included *MYD88* and three uncharacterized loci (*ENSBTAG00000048268*, *ENSBTAG00000050088*, and *ENSBTAG00000053508*), all associated with innate immunity and defense against trypanosome infection. The hot-humid Mursi cattle habitat, savanna grassland with high tsetse fly prevalence [106], likely exerts strong selection pressure on these immune pathways. *MYD88* encodes an adaptor protein activating Toll-like and interleukin-1 receptor signaling, central to immune responses parasites such as *Trypanosoma* spp. [107,108] and *Toxoplasma* spp. [109].

Several candidate genes were detected in both DHETZ and HHETZ, including *DMXL2*, *TNIP3*, and *VEGFC*, reflecting conserved selection related to thermotolerance, oxidative stress resilience, and metabolic efficiency. For instance, *TNIP3*, also known as TNFAIP3-interacting protein 3, is involved in signal transduction and immune regulatory response, which may influence how cells respond to inflammatory stimuli, such as those associated with lipopolysaccharide pathways [110]. Additionally, TNIP3 protein enhances blood circulation and heat dissipation, both of which are critical for maintaining oxygen delivery during heat stress [59].

A distinct vascular regulatory mechanism may have been selected in cattle populations living in the dry-hot and humid-hot environments. *VEGFC*, detected among the shared candidate genes between the two environments, may reflect a conserved selection on angiogenesis and vascular remodeling pathways that will facilitate peripheral blood flow, oxygen delivery, and heat dissipation during thermal stress. In contrast, *WIF1,* identified as a candidate gene by all four selection-scan methods in HHETZ, may indicate environment-specific modulation of Wnt-related angiogenic pathways under humid-hot conditions. Because humid environments reduce evaporative cooling efficiency and may also coincide with higher pathogen and vector pressure, regulation of vascular and immune-related pathways may represent an adaptive balance between thermoregulation, tissue homeostasis, and inflammatory control. This VEGFC/WIF1 contrast should therefore be viewed as a testable physiological hypothesis requiring functional validation [69,111].

Heat stress also compromises reproductive success, necessitating robust genetic adaptations. Here, *DMXL2*, identified in both groups, regulates gonadotropin-releasing hormone (GnRH) and is essential for ovarian follicle development and reproductive efficiency [52]. Similarly, *HFM1,* detected in HHETZ, is required for spermatogenesis and gametogenesis [67,112]. These findings highlight the genetic foundation of reproductive resilience, ensuring fertility under thermal stress.

Several limitations should be considered when interpreting these selection signatures. First, the relatively modest sample sizes, particularly for HHETZ and some Asian zebu breed subgroups, may reduce statistical power and increase the risk of false-positive signals in haplotype-based tests such as iHS and XP-EHH. Second, the very low genetic differentiation between DHETZ and HHETZ (F_ST_ = 0.0063) indicates a close shared ancestry, meaning that subtle differences in selection statistics should be interpreted with caution. Third, demographic processes such as founder effects, population bottlenecks, recent admixture, and background selection can generate genomic patterns that resemble positive selection. Therefore, the candidate genes identified here should be considered putative adaptive loci rather than confirmed causal variants, and priority was given to signals supported by multiple complementary selection-scan methods. Further validation in larger independent cohorts is required. Last but not least, we also acknowledge that thermotolerance may involve additional mechanisms, including epigenetic regulation, copy number variation, and microbiome-mediated effects, which are complementary to but not directly assessed by the SNP-based selection-scan framework used here.

From an applied perspective, the candidate genes identified here should not be used immediately as validated markers for selection. Rather, they provide a prioritized set of loci for follow-up genotyping, association analysis, and functional validation in larger Ethiopian cattle cohorts. If validated, markers linked to oxidative stress tolerance, protein-folding capacity, vascular remodeling, immune resilience, and reproductive stability could inform genomic selection indices for climate-resilient cattle breeding. Conservation programs should also consider maintaining both DHETZ and HHETZ genetic resources, as each group may harbor distinct adaptive variants relevant to future climatic scenarios.

## Conclusions

This study reanalyzed publicly available WGS datasets to investigate selection signatures associated with heat-stress adaptation in indigenous Ethiopian zebu cattle from hot-arid and hot-humid environments. Despite low genetic differentiation between DHETZ and HHETZ, the two groups showed both shared and environment-specific candidate selection signals. DHETZ were characterized mainly by candidate genes linked to oxidative stress regulation, protein folding, mitochondrial function, vascular remodeling, pigmentation, and reproductive resilience, including *SESN2*, *DNAJC8*, *GRPEL2*, *XBP1*, *VEGFC*, *TNIP3*, and *DMXL2*. In contrast, HHETZ showed stronger signals involving immune response, metabolic regulation, and anti-angiogenic pathways, including *MYD88* and *WIF1*. The shared *VEGFC* signal and HHETZ-specific WIF1 signal suggest a potential vascular-regulatory contrast between arid and humid heat adaptation, although this hypothesis requires functional validation. Because this study is based on secondary analysis of publicly available datasets and includes modest sample sizes, the candidate genes should be considered putative adaptive loci rather than confirmed causal markers. Future studies should validate these findings through targeted genotyping in larger independent cohorts, functional characterization in in vitro and in vivo heat-stress models, integration with transcriptomic and phenotypic data, and broader sampling across Ethiopian altitudinal and rainfall gradients. These findings provide a prioritized set of candidate loci for future validation and may support long-term genomic-informed breeding and conservation strategies for climate-resilient African zebu cattle.

## Supporting information

S1 Table List of breed groups, sequence sources (Bioproject and Biosamples), and SNP analysis in the all cattle dataset. (XLSX)

S2 Table Cross-validation error of admixture analysis at different ancestral proportions at K=1 to K=10. (XLSX)

S3 Table Candidate genes detected by the integrated haplotype score (iHS) selection scan method in DHETZ. (XLSX)

S4 Table Candidate genes detected by the pooled heterozygosity (Hp) selection scan method in DHETZ. (XLSX)

S5 Table Candidate genes detected by the integrated haplotype score (iHS) selection scan method in HHETZ. (XLSX)

S6 Table Candidate genes detected by the pooled heterozygosity (*Hp*) selection scan method in HHETZ. (XLSX)

S7 Table Candidate genes detected by the cross-population extended haplotype homozygosity (XP-EHH) selection scan method comparing DHETZ and HHETZ. (XLSX)

S8 Table Candidate genes detected by the cross-population composite likelihood ratio (XP-CLR) selection scan method comparing DHETZ and HHETZ. (XLSX)

S9 Table Candidate gene regions and annotated genes detected by the XP-EHH selection scan method in DHETZ and ASZ cattle. (XLSX)

S10 Table Candidate gene regions and annotated genes detected by the XP-CLR selection scan method in DHETZ and ASZ cattle. (XLSX)

S11 Table Candidate genes detected by the XP-EHH selection scan method in HHETZ and ASZ cattle. (XLSX)

S12 Table Candidate genes detected by the XP-CLR selection scan method in HHETZ and ASZ cattle. (XLSX)

S13 Table The DAVID functional annotation of candidate genes identified by the Hp, iHS, XP-EHH, and XP-CLR selection scan methods in DHETZ. (XLSX)

S14 Table The DAVID functional annotation of candidate genes identified by the Hp, iHS, XP-EHH, and XP-CLR selection scan methods in HHETZ. (XLSX)

S15 Table The DAVID functional annotation of candidate genes identified by the XP-EHH and XP-CLR selection scan methods comparing DHETZ and HHETZ. (XLSX)
