# Supplementary material for: Whole-genome sequencing reveals divergent and shared selection signatures of heat stress adaptation in indigenous Ethiopian zebu cattle from dry-hot and humid-hot environments
Source: PLoS One. 2026 Sep 3;21(9):e0343484. doi: 10.1371/journal.pone.0343484 (PMC13541185; doi:10.1371/journal.pone.0343484)
Supplement: Supplementary file 1 [file pone.0343484.s016.docx]

**Review Report**

**Submitted Title:** “The genomic basis of heat stress adaptation in indigenous Ethiopian cattle from arid and hot-humid climates” (Manuscript Number: PONE-D-26-06464)

**Summary of the Manuscript and Its Key Contributions:** This manuscript investigates the genomic architecture of heat stress adaptation in indigenous Ethiopian zebu cattle (*Bos indicus*) inhabiting two climatically distinct environments: hot-arid lowlands and hot-humid subtropical zones. The study employs whole-genome sequencing (WGS) data from 46 Ethiopian zebu cattle drawn from five populations namly Afar, Ogaden, and Boran (collectively designated as Dry-Heat Ethiopian Zebu, DHETZ) and Mursi and Goffa (designated as Humid-Heat Ethiopian Zebu, **HHETZ**). These populations are compared with Asian zebu (ASZ), Sudanese zebu (SUZ), African taurine (AFT), and European taurine (EUT) cattle. The study integrates multiple complementary genomic approaches: genome-wide single nucleotide polymorphism (SNP) analysis, principal component analysis (PCA), admixture modeling using ADMIXTURE software, and four positive selection scan methods, integrated haplotype score (iHS), pooled heterozygosity (Hp), cross-population extended haplotype homozygosity (XP-EHH), and cross-population composite likelihood ratio (XP-CLR). Functional annotation is performed using Ensembl BioMart and the Database for Annotation, Visualization, and Integrated Discovery (DAVID), with pathway enrichment assessed via Gene Ontology (GO) and Kyoto Encyclopedia of Genes and Genomes (KEGG) databases. The study identifies population-specific and shared genomic selection signatures, reporting environment-specific candidate genes: SESN2, DNAJC8, GRPEL2, ABLIM3, and AFAP1L1 in DHETZ cattle (linked to oxidative stress regulation, protein folding, and vascular remodeling), and MYD88, PRKACA, PRKACB, and WIF1 in **HHETZ** cattle (associated with immune defense, energy metabolism, and angiogenesis inhibition). Genes common to both groups; notably VEGFC, TNIP3, and DMXL2 are proposed as markers of conserved thermotolerance and reproductive adaptation. The key contributions of this work are significant from both scientific and applied perspectives. First, this study presents one of the most comprehensive whole-genome investigations of heat-stress genomics in Ethiopian indigenous cattle, a population that has received comparatively limited attention in the global livestock genomics literature. Second, the comparative framework; contrasting hot-arid versus hot-humid populations within a genetically close population set provides a rigorous and biologically interpretable natural experiment for dissecting climate-specific genomic adaptation. Third, the dual pattern of shared core heat-response pathways and environment-specific metabolic and vascular signatures offers important mechanistic insights that go beyond what phenotypic or single-locus analyses could reveal. Fourth, the identification of candidate marker genes has direct implications for genomic-assisted breeding of climate-resilient livestock under projected future climate change scenarios, particularly in sub-Saharan Africa where heat stress is a growing threat to food security and pastoral livelihoods. Overall, the research question is timely, the dataset is reasonably well-constructed, and the findings add meaningfully to the growing body of livestock population genomics literature. However, as detailed in the sections below, several methodological, analytical, and presentation issues require substantial revision before the manuscript can be considered suitable for publication.

**SECTION WISE EVALUATION**

Title Evaluation: The submitted title is scientifically informative and broadly appropriate for the study. It correctly situates the manuscript within the fields of animal genomics and climate adaptation. However, several improvements are recommended. First, the title would benefit from greater specificity regarding the methodological approach. The phrase “genomic basis” is accurate but vague; incorporating a reference to whole-genome sequencing or selection signatures would better differentiate this study from array-based genotyping studies and signal methodological rigor to readers. Second, the term “indigenous Ethiopian cattle” does not reflect the zebu (*Bos indicus*) classification of the study populations, which is a scientifically important distinction and is foregrounded in the abstract. Including “zebu” in the title would improve its indexability in genomic databases and its relevance for researchers working specifically on indicine cattle biology. A revised title is proposed as follows: “Whole-Genome Sequencing Reveals Divergent and Shared Selection Signatures of Heat Stress Adaptation in Indigenous Ethiopian Zebu Cattle from Hot-Arid and Hot-Humid Environments.” This alternative is more specific, methodologically informative, and terminologically precise. It also avoids the passive construction “genomic basis of,” which could be construed as implying a more mechanistic resolution than WGS selection scans alone can provide.  Abstract Evaluation: The abstract provides a reasonably complete overview of the study's objectives, methodology, and findings and is generally well-structured. Nevertheless, several issues compromise its scientific precision and clarity, and targeted revisions are necessary. Regarding scientific precision: the abstract states that the study “performed whole-genome sequencing of 46 Ethiopian zebu cattle from five populations,” which implies that sequencing was conducted as part of this study. However, the Methods section clarifies that all WGS data were retrieved from publicly available repositories, including NCBI SRA and EMBRAPA. The abstract must be corrected to accurately reflect that WGS data were obtained from public repositories and re-analyzed, rather than implying that original sequencing was performed by the authors. This distinction is critical for transparency and reproducibility, and its omission could mislead readers. Regarding quantitative completeness: the abstract mentions that “Ethiopian zebu closely clustered with Sudanese zebu but showed clear divergence from Asian zebu and taurine breeds,” but does not report any quantitative measures of population differentiation (e.g., FST values) that would allow readers to assess the magnitude of these differences. Similarly, the abstract mentions selection in multiple genes without specifying the number of candidate regions identified per environment or per method. Including at least summary quantitative metrics would substantially improve the abstract's informational value. Regarding novelty framing: the abstract's concluding sentence, “This study provides novel insights into the genomic architecture of environmental adaptation in tropical cattle and offers valuable markers for breeding climate-resilient livestock” is too generic and does not distinguish the findings from prior studies on heat-stress genomics in African cattle. The abstract should conclude with a more specific and compelling statement of what is novel, for instance, the identification of divergent vascular adaptation strategies (VEGFC versus WIF1) as a distinguishing feature of arid versus humid heat stress, or the use of multiple complementary selection statistics in a natural comparative experiment. Minor language issues such as the phrase “the genomic bases of this resilience are not fully elucidated” should be revised to “the genomic basis of this resilience remains incompletely characterized” for greater precision and stylistic consistency. The phrase “remarkably adaptation” in some sentence constructions should be reviewed carefully. Throughout the abstract, passive constructions dominate; selectively replacing these with active voice would improve readability.

**Introduction Evaluation:** The Introduction situates the study within a broad body of literature on African cattle diversity, environmental adaptation, and livestock genomics. It is generally well-organized and progresses logically from general to specific. However, several analytical, structural, and language issues require attention. In terms of literature gaps and justification: the Introduction does a reasonable job of summarizing prior work on heat-stress genomics in cattle but does not clearly articulate what specific gaps this study fills beyond what has already been done. For instance, Terefe et al. (2023); reference [11], which includes some of the present authors, already examined selection signatures in Ethiopian indigenous cattle. The Introduction does not clearly explain how the present study advances beyond this prior work, either in terms of sample size, methodological rigor, the breadth of selection statistics used, or the specific comparative framework employed. A more explicit statement of how this study is distinct from and complementary to the authors' prior work is essential. Regarding the description of Ethiopian cattle ecology: the Introduction correctly notes that Ethiopian zebu are adapted to contrasting environments and describes them as an excellent model for studying thermotolerance genomics. However, the description of the specific agro-ecological zones — hot-arid versus hot-humid, is deferred entirely to the Methods section. Incorporating a brief description of these environments in the Introduction, along with relevant climate data, would better contextualize the biological rationale for the study and help readers unfamiliar with Ethiopian cattle understand the ecological basis of the comparison. Regarding breadth of adaptation mechanisms cited: the Introduction discusses morphological, physiological, and genomic mechanisms of heat adaptation in cattle. However, the coverage is selective. Notable omissions include the role of epigenetic regulation in heat stress response, the contribution of copy number variants (CNVs) to phenotypic plasticity, and the growing recognition that microbiome composition mediates thermotolerance in ruminants. While these topics need not be studied in this manuscript, briefly acknowledging them as complementary avenues would strengthen the literature framing. Language and structural issues like the phrase “broadly into African zebu” in the first paragraph is grammatically incomplete and should read “broadly classified into African zebu.” The sentence “These cattle exhibit distinct morphological and physiological adaptations to extreme environments, including a prominent hump, large ears, loose skin, and variable coat colors” would benefit from a brief explanation of the functional significance of each trait before citing references. Transition sentences between paragraphs are occasionally abrupt, particularly between the paragraph on heat-stress physiology and the one on Ethiopian cattle ecology. Strengthening these transitions would improve the narrative flow. The Introduction's final paragraph, which describes the study's specific objectives, is clear and appropriately scoped. However, it would benefit from explicitly stating the hypothesis being tested; for example, whether the authors hypothesize that DHETZ and HHETZ populations, despite shared ancestry, harbor distinct genomic signatures reflecting divergent adaptive strategies, or whether they hypothesize partially shared and partially divergent selection. Making the study's a priori expectations explicit would allow readers to properly evaluate the significance of the findings.

**Materials and Methods Evaluation:**

**Data sourcing and sample description:** A critical and fundamental concern regarding the Methods section is the discrepancy between the stated sample size and the data sourcing approach. The manuscript states that it analyzed WGS data from 46 Ethiopian cattle drawn from five populations and compared them to 85 additional animals from non-Ethiopian breeds, for a total dataset of 131 animals. All data were retrieved from publicly available repositories (NCBI SRA, CNGB, and EMBRAPA). However, the abstract and introduction imply that sequencing was conducted as part of this study. This should be corrected throughout the manuscript. Transparency regarding data reuse is not only a scientific integrity issue but is also required under PLOS ONE's policies. The authors must clearly state in the Methods that this is a reanalysis of publicly deposited WGS data, and must confirm that all data access was consistent with the original data-sharing terms of each repository. The inclusion of Gir cattle data from EMBRAPA, which is described as restricted-access, requires further clarification. The manuscript states that access is restricted and that data can be shared upon request with the corresponding author at EMBRAPA. Given that PLOS ONE requires full data availability, the editorial team should carefully evaluate whether this restriction is compatible with the journal's data policy. The authors should provide additional documentation of the conditions under which this dataset was accessed and used. Additionally, the sample sizes for some populations are small. The HHETZ group comprises only 18 animals (10 Mursi, 8 Goffa), and some of the Asian zebu populations have as few as two individuals per breed. Small sample sizes in selection scan analyses can inflate false-positive rates, particularly for iHS and XP-EHH, which are sensitive to linkage disequilibrium structure estimated from limited individuals. The authors should explicitly discuss how these sample sizes may affect the power and reliability of selection scan results, and should ideally provide a formal power analysis or cite appropriate literature on minimum sample requirements for the methods used.

**Bioinformatics pipeline:** The bioinformatics pipeline is described with adequate detail for the key processing steps: quality control using Trimmomatic v0.38, alignment to the ARS-UCD1.2 reference genome using BWA-MEM (v0.7.17), base quality score recalibration and variant calling using GATK v3.8, and joint genotyping across all samples. The use of known polymorphisms from the 1000 Bull Genomes Project Run 8 for BQSR is appropriate and well-justified. However, several methodological details are either absent or insufficiently described. First, the duplication marking step is not mentioned. Optical duplicates are a common artifact in Illumina sequencing and must be removed prior to variant calling using tools such as Picard MarkDuplicates or GATK MarkDuplicates. The authors should clarify whether this step was performed and, if not, why it was omitted. Second, the minimum sequencing depth used to include or exclude individual animals is not specified. Shallow sequencing depth can severely bias allele frequency estimation and downstream population genetic statistics. The mean sequencing depth per sample and the depth cutoffs used for variant filtering should be reported, ideally in a supplementary table. Third, regarding variant filtration: the manuscript states that variant quality score recalibration (VQSR) was performed using a 99.9% truth sensitivity filter. However, the training datasets used for VQSR, specifically, the “true” and “false positive” variant sets are not described. For bovine WGS data, appropriate VQSR training sets may differ from those used in human genomics, and the authors should specify which resource files were used. Furthermore, the final hard filters applied to retain only biallelic SNPs, remove indels, and apply site-level quality metrics (e.g., QD, FS, MQ, SOR thresholds) are not described. These details are essential for reproducibility and should be reported in full. Fourth, the LD pruning parameters used for admixture analysis are only partially described: the manuscript states that linked SNPs with LD > 0.5 were removed, but does not specify the window size or step used for LD computation. For PLINK-based LD pruning, these parameters substantially affect the number and independence of retained SNPs and should be reported explicitly.

**Selection scan methodology:** The four selection scan methods namely,  iHS, Hp, XP-EHH, and XP-CLR, represent a reasonable and complementary set of approaches that capture both within-population haplotype-based signatures and cross-population allele frequency differentiation. The use of multiple statistics to identify high-confidence candidates (those detected by at least three of the four methods) is a sound analytical strategy that reduces false positives. The authors are commended for this integrative approach. Nevertheless, several aspects of the selection scan implementation require clarification or improvement. For iHS, the authors state that genotypes were phased and imputed using BEAGLE v5.1 prior to analysis. The use of imputation in this context is potentially problematic: imputation fills in missing genotypes based on a reference panel, and if the reference panel does not adequately represent these Ethiopian zebu populations, imputation could introduce systematic biases in haplotype structure and, by extension, in iHS scores. The authors should specify which reference panel was used for imputation and should justify its appropriateness for the study populations. Alternatively, if BEAGLE was used only for phasing (without imputation of missing genotypes), this should be clarified. For Hp, the manuscript states that the analysis identifies regions of excess heterozygosity across the combined DHETZ and HHETZ genomes, but the mathematical formulation and the specific software or scripts used are not provided. The original Hp statistic, as described by Rubin et al. (2010), is designed for pooled sequencing data rather than individual-level WGS data. If individual-level WGS data were used, the authors should clarify how individual genotypes were pooled and how the Hp statistic was adapted for this data format. For XP-EHH and XP-CLR, the sliding window parameters (100 kb window, 50 kb step size) are stated but not justified. The choice of window size affects the resolution and sensitivity of both statistics, and the rationale for these specific parameters should be provided. Additionally, the software packages used to compute XP-EHH and XP-CLR are not specified; this omission makes the analysis non-reproducible. The authors should cite the specific tools used (e.g., selscan, haplohh, or custom scripts) and provide version numbers. The significance thresholds used for each statistic vary: iHS uses P < 3.96×10⁻⁵ for DHETZ and P = 6.22×10⁻⁶ for HHETZ; Hp uses Z-Hp < −3.3 for DHETZ and Z-Hp < −3.2 for HHETZ; XP-EHH uses P < 1.0×10⁻⁶; and XP-CLR uses an empirical score threshold. The inconsistency in threshold values between DHETZ and HHETZ for the same statistics is not explained and could reflect differences in the statistical distributions due to sample size disparities. The authors should justify all threshold choices, ideally with reference to standard practices or simulation-based calibration.

**Functional annotation:** The use of Ensembl BioMart for gene annotation and DAVID for GO/KEGG enrichment analysis is appropriate and widely accepted in the field. However, the DAVID bioinformatics tool has not been updated since 2016 for many biological databases, and more contemporary alternatives such as g:Profiler or EnrichR provide more current annotations and greater flexibility in background set specification. The authors should either use a more recently updated annotation platform or explicitly justify their use of DAVID and discuss any potential limitations arising from its use of older database versions. Furthermore, the background gene set used for enrichment analysis is not specified. DAVID enrichment results are highly sensitive to the choice of background: using the entire bovine genome as background versus using only the genes in candidate regions as background can lead to dramatically different enrichment outcomes. The authors should clearly state what background was used and should verify that GO and KEGG annotations are available for all candidate genes in the bovine genome.

**Results Evaluation:**

**Variant discovery and population structure:** The SNP summary statistics reported in Table 1 are generally informative and reveal meaningful differences among populations. The observation that HHETZ cattle exhibit higher heterozygosity and lower inbreeding compared to DHETZ cattle is biologically interesting and is appropriately discussed. The PCA results are clearly described, and the clustering of Ethiopian zebu with Sudanese zebu; separated from Asian zebu and taurine breeds, is consistent with known phylogeographic patterns of African cattle. However, several issues with the presentation and interpretation of these results require attention. First, in the PCA results, the authors state that PC1 explained 48.7% of variance and PC2 explained 12.8%, for a combined explained variance of 61.5%. However, Figure 1C, which shows PCA specifically within Ethiopian cattle populations, reports different percentage values (PC1: 11.5%, PC2: 10.5% based on the axis labels visible in the figure). The discrepancy between the overall PCA variance explained and the within-Ethiopian PCA variance explained should be explicitly discussed, as these represent different analyses with different variance structures. This distinction is currently not clearly made in the Results text. Second, the FST values reported in Table 2 are described as having been computed using 20 samples per group, but the HHETZ group contains only 18 animals and the DHETZ group contains 28 animals. The statement that “20 samples per group” were used raises concerns: were animals randomly subsampled to achieve equal group sizes? If so, this sampling approach should be described in the Methods and the potential impact on FST estimates should be discussed. Third, Table 1 contains a typographic error: the total biallelic autosomal SNPs for SUZ are listed as “29,460;635” (with a semicolon instead of a comma). This should be corrected to “29,460,635.” The authors should carefully proofread all tables for similar formatting errors. Fourth, the admixture analysis results are only partially described in the text. The manuscript states that K = 4 is optimal based on the lowest cross-validation error, but Figure 2A shows that the CV error curve does not show a pronounced minimum at K = 4; rather, it shows a gradual decrease from K = 2 to K = 4 followed by a plateau. This pattern may indicate that K = 4 is not unambiguously optimal and that the data are consistent with multiple K values. The authors should provide the actual CV error values for each K in a supplementary table and should discuss the robustness of the K = 4 interpretation.

**Selection signatures:** The Results section for selection signatures is among the most detailed portions of the manuscript but has several presentation and interpretive shortcomings that require revision. The candidate gene tables (Tables 3, 4, and 5) are generally well-organized and informative, listing genomic coordinates, selection statistics, gene names, and phenotypic associations with references. However, the tables contain several errors and inconsistencies that must be corrected. In Table 3 (DHETZ candidates), the chromosomal coordinates are presented inconsistently: some regions are listed in megabase (Mb) units (e.g., “125.10–125.4”), while others appear in base pair or kilobase units (e.g., “573369–721435” for the CPE gene on BTA17). All coordinates should be standardized to a single unit (preferably Mb) for consistency and reader clarity. Additionally, several cells in the HP column of Table 3 contain only dashes (“–”), indicating that the candidate region was not detected by Hp, yet these regions are listed as “identified by at least three methods.” The authors should clarify which three methods detected each region and should consider adding a column explicitly listing the methods that detected each candidate, as this would make the table considerably more interpretable. In Table 3, the reference for TTLL7 is listed as [43], which in the reference list corresponds to a paper by Zhang et al. (2023) on Vav3 expression in myocardial infarction, a topic unrelated to polyglutamylation or TTLL7 function. This appears to be a citation error. Similarly, the reference for SAMD13 is listed as [40], which describes computational identification of heat shock protein genes in the bovine genome, but SAMD13 is not a heat shock protein, and its biological role in heat adaptation is not explained in the text. The authors should verify all citations in the candidate gene tables and replace any erroneous references with appropriate ones. In Table 5 (common candidates between DHETZ and HHETZ), the region start coordinate for BTA1 is listed as “115.5” and the end as “11.6,” which appears to be a typographic error. The end coordinate (11.6 Mb) is smaller than the start coordinate (115.5 Mb), which is physically impossible. This should be corrected. The text discussion of HHETZ selection signatures correctly highlights WIF1 as detected by all four methods, making it the most robustly supported candidate in the HHETZ group. This is an important finding that deserves greater emphasis. The manuscript also correctly notes the association of MYD88 and associated uncharacterized loci with trypanosomiasis resistance, reflecting the high tsetse fly prevalence in the Mursi cattle habitat. These observations are biologically compelling and represent some of the most interesting findings of the study. However, the Results section does not discuss negative results or regions where selection scans failed to converge across methods. Reporting the overlap rates among methods (e.g., what proportion of iHS-detected regions were also detected by XP-EHH or XP-CLR) would provide a useful calibration of statistical consistency and would help readers assess the robustness of the candidate selection approach.

**Discussion Evaluation:** The section is the strongest section of the manuscript in terms of scientific depth and is generally well-written. The authors successfully integrate genomic findings with functional biology, linking specific candidate genes to well-established physiological mechanisms of heat stress response. The discussion of SESN2 as an antioxidant regulator, GRPEL2 as a mitochondrial chaperone, XBP1 as an unfolded protein response mediator, and MYD88 as an innate immune adaptor is scientifically sound and well-referenced. Nevertheless, several areas of the Discussion require strengthening. First, the Discussion does not explicitly compare findings with results from the authors' prior study (Terefe et al., 2023, reference [11]), which analyzed selection signatures in overlapping Ethiopian cattle populations. Given that both studies use some of the same populations and some of the same selection statistics, readers will naturally ask whether the candidate genes identified in the present study are consistent with, complementary to, or divergent from those reported previously. The absence of this comparison is a significant analytical gap that the authors must address. Second, the Discussion would benefit from a more explicit treatment of alternative explanations for the observed selection signals. Selection scan statistics such as iHS, Hp, XP-EHH, and XP-CLR cannot distinguish between positive selection and other demographic processes that can produce similar genomic signatures, including population bottlenecks, founder effects, population structure, and background selection. Given the limited genetic differentiation between DHETZ and HHETZ (FST = 0.0063), the concern that some apparent selection signals may reflect shared demographic history rather than environment-specific adaptation is particularly relevant. The authors should discuss this limitation more explicitly and, if possible, reference appropriate null demographic models or simulation-based calibration approaches. Third, the discussion of common candidate genes (DMXL2, TNIP3, VEGFC) is relatively brief given their potential significance as shared thermotolerance markers across both environments. The functional role of DMXL2 in regulating GnRH secretion and ovarian follicle development is discussed, but its relevance to heat stress, which is known to disrupt the hypothalamic-pituitary-gonadal axis, could be elaborated further. Similarly, the opposing roles of VEGFC (pro-angiogenic, shared between **DHETZ** and **HHETZ**) and WIF1 (anti-angiogenic, specific to **HHETZ**) represent a particularly elegant biological finding that deserves more detailed mechanistic interpretation. Why would hot-humid environments favor anti-angiogenic signaling while both environments select for pro-angiogenic **VEGFC**? A more developed physiological hypothesis addressing this question would substantially strengthen the manuscript. Fourth, the Discussion does not adequately address the practical implications of the findings for livestock improvement programs. The concluding paragraph of the Discussion briefly mentions “genomic-informed breeding programs” but does not specify how the identified candidate markers could be integrated into genomic selection indices, marker-assisted selection programs, or conservation genomics priorities. Given that one of the study's stated objectives is to identify valuable markers for breeding climate-resilient livestock, this gap between findings and application should be addressed more concretely. Fifth, the sentence in the Discussion that reads “TNFAIP3 (TNIP3 interacting protein 3) is involved in signal transduction and immunoregulatory” is grammatically incomplete (missing a predicate complement after “immunoregulatory”) and also contains a nomenclature inaccuracy: TNIP3 is TNFAIP3-interacting protein 3, not TNFAIP3 itself. These should be corrected.

**Conclusions Evaluation:** The Conclusions section is brief and largely recapitulates points already made in the abstract and discussion. While it correctly identifies the dual pattern of shared and environment-specific genomic adaptation as the central finding, it does so at a level of generality that does not convey the specific novelty of the study. The conclusion should be expanded to include explicit statements of: (a) the most robust candidate genes and their functional significance; (b) the specific contrast between arid-adapted (oxidative stress, protein folding, mitochondrial function) and humid-adapted (immune response, energy metabolism, anti-angiogenesis) genomic profiles; (c) the convergent vascular selection findings (VEGFC versus WIF1); and (d) specific recommendations for how these findings can inform breeding or conservation programs. The concluding call for future research integrating functional genomics and transcriptomics is appropriate and important. However, the conclusion should also recommend population-level validation of identified candidate variants through targeted genotyping in larger cohorts, functional characterization of top candidates in in vitro or in vivo heat stress models, and investigation of gene-environment interaction effects across broader Ethiopian cattle populations spanning altitudinal and rainfall gradients. Furthermore, the limitation that all data were sourced from public repositories, and that the study is therefore a reanalysis rather than an original sequencing effort, should be acknowledged in the Conclusions as a boundary condition on the study's claims. The conclusion should also acknowledge that selection scan methods identify genomic regions rather than causal variants, and that fine-mapping and functional studies will be required to resolve specific causal mutations within the identified candidate regions.

**Language, Grammar, and Style Improvements:** The manuscript is written in English but contains numerous grammatical errors, awkward constructions, and stylistic inconsistencies that require thorough correction. The following are representative examples, and the authors are strongly encouraged to seek professional English language editing before resubmission. In the abstract, “exhibit remarkable adaptation to extreme thermal conditions” should read “exhibit remarkable adaptations to extreme thermal conditions” (plural). The phrase “yet the genomic bases of this resilience are not fully elucidated” is grammatically correct but is a passive construction that could more naturally read “yet the genomic basis of this resilience remains incompletely characterized.” The phrase “by integrating genome-wide SNP analysis, population genetic structure assessment, and multiple selection scans” would read better as “by integrating genome-wide SNP analysis, population-level genetic structure assessment, and four complementary selection scan methods.” In the Introduction, “broadly into African zebu” (first paragraph) should read “broadly classified into African zebu.” The phrase “rendering zebu cattle highly suited to tropical and arid climates” lacks a subject in its participial phrase and should be revised to read “rendering zebu cattle highly suited to life in tropical and arid climates” or restructured as a full clause. “Heat stress adaptation in tropical cattle involves an integrated suite of morphological, behavioral, physiological, neuroendocrine, and metabolic modifications” — while scientifically correct, this sentence would benefit from a supporting citation to a comprehensive review. In the Methods section, “Quality control of raw sequence reads was performed using Trimmomatic v0.38, removing short reads (< 35 bp) and low-quality bases (Phred score < 20)” should read “...by removing short reads shorter than 35 bp and bases with a Phred quality score below 20.” The phrase “following best practices recommendations” should specify which best practices document is meant (GATK Best Practices), preferably with a citation. “Variants of each sample were identified using the GATK HaplotypeCaller” is correct but imprecise; it should clarify that HaplotypeCaller was run in GVCF mode for joint calling. In the Results section, “The HP scan identifies 113 non-overlapping candidate genome regions” inconsistently uses the present tense alongside past tense verbs in adjacent sentences; all Results text should be uniformly in past tense. The expression “overlapping with 183 protein-coding genes” would read better as “encompassing 183 protein-coding genes.” The phrase “Ethiopian Zebu groups (DHETZ and HHETZ) display comparable SNP counts, despite inhabiting contrasting agro-ecologies (dry-hot vs. hot-humid)”, the shift to present tense here is inconsistent and should be corrected. In the Discussion, “Heat stress can disrupt protein folding, cause cellular damage, impair metabolism, and impact reproductive physiology” is a correct sentence but uses “impact” as a verb (acceptable in scientific English but sometimes considered informal); “affect” would be preferable. “To mitigate these effects, molecular chaperones and stress regulators play essential roles” — the subject of the infinitive phrase is unclear; restructuring as “Molecular chaperones and stress regulators mitigate these effects by maintaining protein stability and cellular homeostasis” would be clearer. The phrase “thermally adapted livestock populations” at the end of the Conclusions section should be “thermally adapted livestock” (“populations” is implied and redundant). Several instances of potential artificial intelligence-generated phrasing are present in the manuscript, including repetitive sentence structures, overuse of phrases such as “plays a crucial role in,” “is involved in,” and “has been shown to,” and formulaic transitions between subsections. The authors are strongly advised to revise the manuscript for originality of expression, varying sentence structure, and eliminating stock phrases that reduce the distinctiveness of the scientific writing. Similarly, the manuscript should be carefully reviewed for self-plagiarism relative to the authors' prior publications, particularly Terefe et al. (2023, reference [11]), and any overlapping text must be appropriately paraphrased, cited, or attributed.

**Figures and Tables Evaluation:** The manuscript includes six figures and five in-text tables (Tables 1–5), plus 14 supplementary Excel files. Overall, the figures are scientifically appropriate for the analyses performed, but several presentation issues require attention. Regarding Figure 1 (PCA plots): the color coding and population labels in panels A and B are small and may be difficult to distinguish in print. The font sizes in the axis labels are inconsistently sized across panels A, B, and C. The legend labels are abbreviations (AFT, ASZ, DHETZ, EUT, HHETZ, SUZ) that should be defined in the figure legend. Panel C uses a different set of population labels (Afar, ET-Boran, Goffa, Mursi, Ogaden), which are appropriate for within-Ethiopian comparison but should be cross-referenced with the DHETZ/HHETZ groupings in the legend. The axes of panel C show PC1 at 11.5% and PC2 at 10.5%, but the text reports PC1 at 48.7% and PC2 at 12.8% for the overall PCA. This is presumably because panel C is a separate analysis restricted to Ethiopian cattle, but the figure legend does not make this distinction explicit. Regarding Figure 2 (Admixture plots): the CV error curve in panel A is clearly presented. However, in panel B, the population labels along the X-axis (HHETZ, DHETZ, SUZ, ASZ, AFT, EUT) do not include sample sizes, which would help readers assess the width of each population bar relative to sample size. The color scheme in the admixture bars should be defined in the figure legend with explicit descriptions of what each color represents at each K value. Regarding Figures 3–6 (Manhattan plots): these are generally well-executed and appropriately labeled. The gene annotations on the Manhattan plots are legible and correctly positioned. However, the statistical threshold lines (dotted horizontal lines) should be explicitly defined in the figure legends with the exact threshold values used, rather than requiring the reader to cross-reference the Methods section. For Hp plots (Figures 3B, 4B), the Y-axis is inverted (negative ZHp values indicate selection), and this should be explicitly stated in the figure legend to avoid misinterpretation. Regarding Table 3 (DHETZ candidates): as noted in the Results evaluation above, the chromosomal coordinates in Table 3 are presented in inconsistent units. Additionally, the column headers “Reg. Start” and “Reg. End” should be expanded to “Region Start (Mb)” and “Region End (Mb)” for clarity. The columns for HP, iHS, XP-EHH, and XP-CLR should include a column header footnote specifying the units of each statistic (Z-score for HP, standardized iHS score, -log10 P-value for XP-EHH, and raw score for XP-CLR). Dashes indicating non-detection by a given method should be replaced with “N.D.” (Not Detected) for professional clarity.

**References Evaluation:** The reference list is comprehensive and generally appropriate for the scope of the study. The manuscript cites 109 references, including both seminal methodological papers and recent genomics literature. However, several reference-related issues require attention. First, citation errors are present in the candidate gene tables, as noted in the Results evaluation above. The reference for TTLL7 in Table 3 (reference [43]: Zhang et al., 2023, on Vav3 in myocardial infarction) does not correspond to the functional annotation given (protein modification/polyglutamylation in the central nervous system). The authors should verify every citation in the candidate gene tables against the stated phenotype or function. Second, some references appear to be cited for general functional categories rather than specifically for the candidate gene or phenotype in question. For example, reference [43] in Table 3 (cited for TTLL7) should be replaced with a reference specifically describing TTLL7's role in polyglutamylation. Similarly, the reference for AFAP1L1 in the Discussion (reference [94]: Wang et al., 2018) pertains to non-small cell lung cancer rather than to heat stress or muscle physiology, while AFAP1L1's role in cell proliferation is relevant, a more directly relevant reference should be sought if available. Third, the manuscript cites reference [82] (Wolf et al., 2021) with a note “[cited 6 Aug 2025],” which is unusual for a journal article citation and suggests this may be a preprint or conference paper accessed online. The authors should verify the publication status of this reference and update the citation accordingly. Fourth, a small number of references appear duplicated in function: references [107] and [108] both cite Chen et al. (2016) in Reproductive Sciences on WIF1 under hypoxic conditions. These appear to be the same article cited twice in the same context. The authors should verify and remove duplicate citations.

**Data Availability and Ethics Statement:** The data availability statement indicates that some data have restricted access, specifically the Gir cattle WGS data from EMBRAPA. This restriction is potentially incompatible with PLOS ONE's data sharing policy, which requires that data underlying published findings be fully available without restriction. The authors and the editorial team should resolve this issue prior to acceptance. A possible resolution would be to exclude the Gir data from the main analyses and conduct a sensitivity analysis to determine whether its inclusion materially affects the principal conclusions, or alternatively to obtain written documentation from EMBRAPA confirming that the data can be released upon acceptance. The manuscript's ethics statement is listed as “N/A” in the submission form, presumably because the study involves reanalysis of publicly deposited genomic data from animals that were sampled by others. This position is defensible provided that the original data collection was conducted under appropriate ethical oversight. However, the authors should explicitly state in the Methods section that this is a secondary analysis of previously published and/or publicly deposited data, and should note that the original sample collection ethics are covered by the primary data sources.

**Constructive Feedback and Recommendations for the Authors:** The following prioritized recommendations are offered in the spirit of constructive scientific dialogue. The authors have produced a study with genuine scientific value, and the recommendations below are intended to help them bring the manuscript to the standard required for publication in a high-quality peer-reviewed journal. Critical (must be addressed prior to acceptance): (1) Correct the misrepresentation of original WGS data generation — make explicit throughout the manuscript that publicly available WGS data were reanalyzed. (2) Resolve the EMBRAPA restricted-access data issue in compliance with PLOS ONE data policy. (3) Specify the LD pruning parameters for admixture analysis, the software and version numbers for XP-EHH and XP-CLR computation, the duplication-marking protocol, and the VQSR training datasets. (4) Correct the chromosomal coordinate inconsistencies and citation errors in Tables 3, 4, and 5. (5) Compare findings explicitly with the prior Terefe et al. (2023) study and clarify the incremental contribution of the present work. (6) Conduct professional English language editing throughout the manuscript. Major (should be addressed prior to acceptance): (7) Provide a discussion of the potential impact of small sample sizes on selection scan reliability, ideally with a power analysis or citation to relevant methodological literature. (8) Justify the BEAGLE imputation approach and reference panel used for iHS phasing. (9) Replace DAVID with a more current enrichment analysis platform, or explicitly justify continued use of DAVID. (10) Specify the background gene set used for GO/KEGG enrichment analysis. (11) Elaborate on the mechanistic interpretation of the VEGFC versus WIF1 contrast as representing divergent vascular adaptation strategies. (12) Strengthen the Conclusions section with specific findings, limitations, and concrete recommendations for application in livestock breeding programs. Minor (recommended for improvement): (13) Revise the title to reflect whole-genome resequencing and zebu taxonomy. (14) Update the abstract to accurately reflect data sourcing and include key quantitative metrics. (15) Standardize units in all tables and define all abbreviations in figure legends. (16) Correct all grammatical errors and inconsistencies in tense usage. (17) Correct the duplicate WIF1 reference and the Wolf et al. (2021) citation status. (18) Add sample sizes to the admixture plot X-axis labels and threshold definitions to Manhattan plot legends.

**Overall Recommendation:** Major revision before acceptance.

This manuscript addresses an important and timely scientific question, employs a broadly sound methodological framework, and reports findings of genuine relevance to both livestock genomics and climate adaptation biology. The study's comparative design, leveraging genetically related Ethiopian zebu populations inhabiting divergent climatic environments, is scientifically elegant, and the integrative multi-method selection scan approach is commendable. However, the manuscript currently has significant methodological documentation gaps, several factual and citation errors, inconsistencies in data presentation, and language issues that together prevent publication in its current form. The most critical concern is the apparent misrepresentation of publicly available data as originally generated WGS data, which must be corrected. Additionally, the data availability issue concerning the restricted EMBRAPA dataset must be resolved in accordance with PLOS ONE policy. Accordingly, the recommendation is Major Revision. The authors are encouraged to prepare a detailed point-by-point response to all concerns raised, with clear indications of where revisions have been made in the manuscript. Upon resubmission, the revised manuscript should be checked by a professional science editor for English language quality. If the authors address all critical and major concerns satisfactorily, the manuscript has strong potential for publication in PLOS ONE.
