# Supplementary material for: Whole-genome sequencing reveals divergent and shared selection signatures of heat stress adaptation in indigenous Ethiopian zebu cattle from dry-hot and humid-hot environments
Source: PLoS One. 2026 Sep 3;21(9):e0343484. doi: 10.1371/journal.pone.0343484 (PMC13541185; doi:10.1371/journal.pone.0343484)
Supplement: Supplementary file 2 [file pone.0343484.s018.docx]

Response to the reviewer

Title: The genomic basis of heat stress adaptation in indigenous Ethiopian cattle from arid and hot-humid climates

Number: PONE-D-26-06464R1

We would like to express our sincere appreciation for the reviewer’s thorough evaluation of our manuscript. The insights and constructive feedback provided were invaluable in enhancing the quality of our work. We truly commend the reviewer for their diligence and thoughtful suggestions. We have carefully considered each comment and made the necessary revisions to address all the points raised. Thank you once again for the time and effort invested in reviewing our manuscript.

Reviewer #1: Section-Wise Evaluation
Title: The title is informative but vague. “Genomic basis” does not distinguish this study from array-based genotyping work, and “indigenous Ethiopian cattle” omits the scientifically important Bos indicus (zebu) classification. A revised title is proposed: “Whole-Genome Sequencing Reveals Divergent and Shared Selection Signatures of Heat Stress Adaptation in Indigenous Ethiopian Zebu Cattle from Hot-Arid and Hot-Humid Environments.” This is more specific, methodologically precise, and avoids implying mechanistic resolution beyond what selection scans can provide.

**Response**: The title has been revised on the title page to specify the use of whole-genome sequencing data, the Bos indicus/zebu classification, and the comparative hot-arid versus hot-humid framework.

Abstract: The abstract is generally well-structured but has three key problems. First, it implies that sequencing was conducted as part of this study, whereas all WGS data were retrieved from public repositories (NCBI SRA, CNGB, EMBRAPA). This must be corrected throughout. Second, it lacks quantitative metrics: no FST values are cited for population differentiation, and no counts of candidate regions per method or environment are reported. Third, the concluding novelty statement is generic; a more specific formulation e.g., highlighting the VEGFC vs. WIF1 vascular divergence, would better distinguish the study. Minor language issues (e.g., “remarkably adaptation,” passive-heavy constructions) also require correction.

**Response**: The Abstract has been revised to clarify that the study is a secondary reanalysis of publicly available whole-genome sequencing datasets rather than newly generated sequencing data. We also added quantitative results, including the number of biallelic autosomal SNPs identified in DHETZ and HHETZ cattle, the low genetic differentiation between DHETZ and HHETZ (FST = 0.0063), the number of candidate regions detected by iHS, Hp, XP-EHH, and XP-CLR, and the number of high-confidence candidate regions supported by multiple selection-scan methods. The concluding sentence was also revised to emphasize the VEGFC/WIF1 vascular-regulatory contrast between arid and humid heat adaptation.

Introduction: The Introduction is logically organized but does not clearly articulate how this study advances beyond the prior work of Terefe et al. (2023, ref. [11]), which includes some of the same authors and overlapping populations. This distinction is essential. Additional gaps include: (a) the agro-ecological description of hot-arid vs. hot-humid zones is deferred entirely to Methods; a brief overview here would better contextualize the biological rationale; (b) notable mechanisms such as epigenetic regulation, copy number variants (CNVs), and microbiome-mediated thermotolerance are unacknowledged as complementary avenues; (c) the final paragraph describing objectives should include an explicit a priori hypothesis (e.g., whether divergent or partially shared selection is expected given low FST). Language issues: “broadly into African zebu” should read “broadly classified into African zebu”; transition sentences between paragraphs on heat-stress physiology and Ethiopian cattle ecology are abrupt.
Materials and Methods:

**Response**: We thank the reviewer for this detailed and constructive comment. We agree that the Introduction needed to distinguish the present study more clearly from Terefe et al. (2023), provide a stronger biological context for the hot-arid and hot-humid comparison, acknowledge complementary mechanisms of thermotolerance, and present a clearer hypothesis. We have revised the Introduction accordingly.

**Revision made:** First, we corrected the language in the opening paragraph by replacing “broadly into African zebu” with “broadly classified into African zebu.” Second, we added a transition sentence linking general heat-stress physiology with the specific ecological setting of Ethiopian cattle. Third, we added a brief agro-ecological overview of the hot-arid and hot-humid environments in the introduction, so that the biological rationale is introduced before the detailed description in Materials and Methods. Fourth, we added a new paragraph clarifying how the present study advances beyond Terefe et al. (2023). Specifically, we now state that while Terefe et al. (2023) described population structure, admixture, and genomic diversity in Ethiopian cattle, the present study focuses specifically on shared and environment-specific selection signatures associated with contrasting thermal and ecological pressures. Fifth, we added a sentence acknowledging that epigenetic regulation, copy number variation, and microbiome-mediated mechanisms may also contribute to thermotolerance but are outside the scope of the current SNP-based selection-scan analysis. Finally, we revised the final paragraph of the Introduction to include an explicit a priori hypothesis that, given the low genetic differentiation between DHETZ and HHETZ cattle, these populations would show both shared thermotolerance-related signatures and environment-specific adaptive signals linked to hot-arid versus hot-humid conditions.

Data sourcing: The critical concern is that all WGS data were retrieved from public repositories, yet the abstract and introduction imply original sequencing. This must be corrected throughout for transparency and compliance with PLOS ONE data policies. The inclusion of restricted-access Gir cattle data from EMBRAPA must be resolved, either by excluding these animals and assessing impact, or by obtaining documented permission for release upon acceptance. The HHETZ group has only 18 animals, and some ASZ populations have as few as two individuals per breed. The authors must discuss how these small sample sizes may inflate false-positive rates for iHS and XP-EHH, ideally with a formal power analysis or citation to relevant literature.
Bioinformatics pipeline: Several critical details are absent: (1) duplication marking (e.g., Picard/GATK MarkDuplicates) is not mentioned; (2) sequencing depth per sample and depth cutoffs for filtering are unspecified; (3) VQSR training datasets for bovine WGS are not described, nor are the hard filters applied (QD, FS, MQ, SOR thresholds); (4) LD pruning parameters for ADMIXTURE (window size and step) are incomplete, only the threshold (r² > 0.5) is stated.

**Response**: We thank the reviewer for highlighting these important concerns regarding data transparency, PLOS ONE data availability requirements, and interpretation of selection-scan results. We agree that the original wording may have unintentionally implied that whole-genome sequencing was newly generated as part of the present study. We have corrected this throughout the manuscript to make clear that this study represents a secondary reanalysis of previously generated whole-genome sequencing datasets obtained from public repositories.

**Revision made**: The Abstract, Introduction, Materials and Methods, Data Availability statement, Ethics statement, and Conclusion have been revised to state explicitly that no new animal sampling, experimental procedures, or sequencing were performed in the present study. Phrases such as “we performed whole-genome sequencing” and “we analyzed WGS data” have been revised where appropriate to “we reanalyzed publicly available whole-genome sequencing datasets”.

**Revision made:** A new subsection titled “Data Sources and Ethics Statement” has been added at the beginning of the Materials and Methods section. This subsection clarifies that the study is based on a secondary analysis of previously generated whole genome sequencing (WGS) datasets. It also notes that ethical approval and sample collection procedures were addressed by the original studies that produced these datasets.

**Revision made:** The Data Availability statement has been revised to clarify the source and accessibility of all whole genome sequencing (WGS) datasets used in this study. To address the issue regarding the restricted-access EMBRAPA Gir cattle dataset, we have obtained permission from Dr. Marcos Vinícius Gualberto Barbosa da Silva ([marcos.vb.silva@embrapa.br](mailto:marcos.vb.silva@embrapa.br) ), one of the co-authors. He has submitted the WGS data of Gir cattle to the NCBI Sequence Read Archive, and the submission is completed with BioProject accession number PRJNA1493839. The BioProject accession number is included in the Data Availability section and in S1 Table. This clarification has been added to the revised Materials and Methods and Data Availability statements.

**Revision made**: We have also added a limitations paragraph to the Discussion addressing the modest sample sizes, including the HHETZ group and some ASZ breed subgroups. We now acknowledge that small sample sizes may reduce statistical power and increase the risk of false-positive signals in haplotype-based selection scans such as iHS and XP-EHH. To reduce overinterpretation, we prioritized only candidate regions supported by multiple complementary selection-scan methods and described these loci as putative adaptive candidates requiring validation in larger independent cohorts.

**Revision made**: Thank you for the suggestions on the Bioinformatics pipeline employed in the analysis, and corrections were done as follows: (1) After the alignment of sequence data to the reference genome (mapping), duplicate reads were identified and marked using the *markDuplicate* option of Picard software Picard v2.18.2. (2) The Variant Quality Score Recalibration (VQSR) was performed using the GATK VariantRecalibrator, utilizing known variant sources from dbSNP of the Bos taurus reference genome obtained from the Ensembl database (version 150). A tranche sensitivity threshold between 99.9% and 100.0% was applied to assess the accuracy of variant mapping to the reference genome. Ultimately, a truth sensitivity threshold of 99.9% was used to filter the variants. Since we use tranche-sensitive truth filtering, we did not employ hard filtering methods. Besides, all the pipelines we used here were obtained from online sources, which we duly acknowledge.

Selection scan methods: The use of BEAGLE for phasing/imputation is problematic if the reference panel does not adequately represent Ethiopian zebu populations, which could introduce biases in iHS haplotype scoring. The Hp statistic, originally designed for pooled sequencing, is applied to individual-level WGS data without explanation of adaptation. For XP-EHH and XP-CLR, the sliding window rationale (100 kb window, 50 kb step) is not justified, and the specific software packages and versions used are not cited; making the analysis non-reproducible. The inconsistent significance thresholds between DHETZ and HHETZ for the same statistics are unexplained.

**Response**: We thank the reviewer for this important comment. We agree that the selection-scan methods require additional clarification to improve reproducibility and to avoid overinterpretation of haplotype-based results. We have expanded the Materials and Methods section to provide additional details on phasing/imputation, the use of Hp with individual-level WGS data, the rationale for the sliding-window approach, the software and versions used for XP-EHH and XP-CLR, and the basis for the significance thresholds applied in DHETZ and HHETZ analyses.

**Revision made**: The “Identification of selection signatures” subsection has been expanded. We now clarify that BEAGLE v5.1 was used for genotype phasing and imputation before iHS analysis, and we acknowledge that the limited availability of Ethiopian zebu-specific reference panels may influence haplotype-based statistics. Therefore, we prioritized candidate regions supported by multiple complementary selection-scan methods rather than relying on iHS alone. We also added an explanation of how Hp was calculated from individual-level WGS data by summarizing heterozygosity across genomic windows, and we clarified that reduced heterozygosity was interpreted as one line of evidence for candidate selective sweeps. In addition, we added the software packages, citations and versions used for XP-EHH and XP-CLR, justified the 100 kb window and 50 kb step size, and explained the empirical/statistical basis for the significance thresholds used in each group. The inconsistent threshold levels in the DHETZ and HHETZ groups arose from the cut point at the top 0.5% of the windows/candidate regions for each selection scan method.

Functional annotation: DAVID has not been updated since 2016; more current platforms (g:Profiler, EnrichR) should be used or DAVID’s limitations explicitly acknowledged. The background gene set for GO/KEGG enrichment is not specified, which significantly affects enrichment outcomes.

**Response**: We appreciate the reviewer's valuable recommendation. We acknowledge that the functional annotation and enrichment analysis needed further clarification, specifically concerning the enrichment platform and the background gene set, as well as an update on the platform used. We have revised the functional annotation subsection to enhance the reproducibility and interpretation of the enrichment results. The online DAVID bioinformatics tool has been regularly updated, and the current version, which we used to annotate the candidate genes, is 2025 (Sherman et al., 2022) (<https://davidbioinformatics.nih.gov/documentation/index.html#release>). However, we cited the initially licensed version of the tool (Huang et al., 2009) in the submitted manuscript. Therefore, we have updated the citation to the current version (Sherman et al., 2022). All protein-coding genes identified within the genomic windows from each selection scan analysis were annotated in the ARS-UCD1.2 genome assembly. The *Bos taurus* genome background was utilized for the functional annotation (GO and KEGG pathways) of these protein-coding genes. Genes that clustered in Gene Ontology (GO) and KEGG pathway terms, with an enrichment fold greater than 1.0 and a Fisher test p-value of less than 0.05, were selected as putative functions for the genes in those clusters.

Results
Variant discovery and population structure: Table 1 contains a typographic error: SUZ biallelic SNPs are listed as “29,460;635” (semicolon) should be “29,460,635.” The FST analysis states “20 samples per group” were used, but HHETZ has only 18 and DHETZ has 28 animals; any subsampling must be described and its effect discussed. The admixture CV error curve does not show a clear minimum at K=4; rather a gradual decrease followed by a plateau, and actual CV error values should be provided in a supplementary table. The PCA discrepancy between overall variance explained (PC1: 48.7%, PC2: 12.8%) and within-Ethiopian variance (PC1: 11.5%, PC2: 10.5%) is not explained; these represent separate analyses and the distinction should be made explicit.

**Response**: We thank the reviewer for identifying these important issues in the Results section. We have corrected the typographical error in Table 1 and revised the FST description to clarify the exact sample numbers used in the population differentiation analysis. We have also revised the ADMIXTURE results to avoid overstatement of K = 4 as a clear optimum and now describe the cross-validation pattern more cautiously. In addition, the actual cross-validation error values for K = 2 to K = 10 have been added as a supplementary table. Finally, we have clarified that the PCA percentages reported for the full and Ethiopian-only datasets correspond to two separate PCA analyses and are therefore not directly comparable.

**Revision made:** Table 1 has been corrected by replacing “29,460;635” with “29,460,635.” The FST was calculated using a random subsample of 20 individuals from each population that had more than 20 members to minimize sampling differences. However, for the HHETZ population, we included all 18 individuals since this number is close to 20. The ADMIXTURE paragraph has been revised to state that the CV error decreased gradually and reached a plateau around K = 4 rather than showing a sharply defined minimum. The CV error values for K = 2 to K = 10 have been added to the Supplementary Table (S2 Table). The PCA Results and Figure 1 legend have been revised to clarify that panels A/B and panel C represent separate analyses: the first across all cattle populations and the second restricted to Ethiopian cattle.

Selection signatures: Candidate gene tables (3–5) contain errors: (1) the TTLL7 reference in Table 3 (ref. [43], Zhang et al. 2023 on Vav3 in myocardial infarction) is unrelated to polyglutamylation and appears misassigned; (2) Table 5 lists a BTA1 region with start 115.5 Mb and end 11.6 Mb; a physically impossible coordinate; (3) chromosomal coordinates in Table 3 are inconsistently presented in Mb and base pairs and must be standardized. The results do not report method overlap rates (e.g., what proportion of iHS-detected regions were confirmed by XP-EHH or XP-CLR), which would help assess statistical consistency. The detection of WIF1 by all four methods in HHETZ; the most robustly supported finding deserves greater emphasis.

**Response:** We thank the reviewer for carefully checking the candidate gene tables and selection-scan results. We agree that these errors and inconsistencies need to be corrected. We have revised Tables 3–5 to standardize chromosomal coordinates, correct the physically impossible coordinate in Table 5, and correct or replace the misassigned TTLL7 reference. We have also revised the Results section to report method-overlap information and to clarify how high-confidence candidate regions were defined. In addition, we have placed stronger emphasis on WIF1 as the most robust HHETZ-specific candidate because it was detected by all four selection-scan methods.

**Revision made:** Tables 3–5 have been corrected and standardized. All chromosomal coordinates are now presented consistently in megabases (mb). Column headings have been revised from “Reg. Start” and “Reg. End” to “Region Start (Mb)” and “Region End (Mb).” Dashes have been replaced by “N.D.” for “Not Detected,” and table footnotes have been added to define each selection statistic and its units. The TTLL7 citation has been corrected, and the erroneous BTA1 coordinate in Table 5 has been fixed. The Results section now reports the number of candidate genes or regions shared across methods and clarifies that high-confidence candidates were prioritized when supported by at least three of the four selection-scan methods. The HHETZ Results section has also been revised to emphasize WIF1 as the only candidate detected by all four methods.

**Revision made:** The “Overlap selection scans candidate genes and functional annotation” subsection has been revised to report how candidate regions were prioritized based on overlap across iHS, Hp, XP-EHH, and XP-CLR. We added that loci supported by at least three of the four methods were considered high-confidence candidates, whereas loci detected by only one method were interpreted cautiously. The HHETZ Results subsection was also revised to emphasize WIF1 as the most consistently supported HHETZ-specific signal because it was detected by all four selection-scan methods.

Discussion: The Discussion is the strongest section, effectively integrating candidate gene biology with heat stress physiology (SESN2/antioxidant regulation, GRPEL2/mitochondrial chaperoning, XBP1/UPR, MYD88/innate immunity). However, several gaps remain: (1) No explicit comparison with Terefe et al. (2023) is made, which is a significant analytical omission given the overlapping populations. (2) Alternative explanations for selection signals population bottlenecks, founder effects, background selection are underexplored, particularly important given the low FST (0.0063) between DHETZ and HHETZ. (3) The biologically elegant contrast of VEGFC (pro-angiogenic, shared) vs. WIF1 (anti-angiogenic, HHETZ-specific) deserves a developed physiological hypothesis. (4) Practical implications for genomic selection indices or marker-assisted selection programs are vague; this should be made concrete. (5) The sentence “TNFAIP3 (TNIP3 interacting protein 3) is involved in signal transduction and immunoregulatory” is grammatically incomplete and contains a nomenclature error: TNIP3 is TNFAIP3-interacting protein 3, not TNFAIP3.

**Response**: We thank the reviewer for the positive assessment of the Discussion and for identifying areas where it could be strengthened. We have revised the Discussion substantially to address each of these points. Specifically, we added an explicit comparison with Terefe et al. (2022 and 2023), expanded the limitations section to discuss demographic processes that can mimic selection, developed the VEGFC/WIF1 vascular-regulatory hypothesis, clarified practical implications for genomic selection and conservation, and corrected the TNIP3 nomenclature and grammar.

**Revision made**: A new paragraph has been added to the Discussion comparing the present study with Terefe et al. (2023). We now clarify that the previous study primarily addressed population structure, admixture, and genomic diversity, as well as high altitude adaptation, whereas the present study focuses on shared and environment-specific selection signatures associated with contrasting hot-arid and hot-humid environments. We also added a limitation paragraph acknowledging that the low FST between DHETZ and HHETZ, together with potential founder effects, bottlenecks, admixture, and background selection, may influence selection-scan signals. The VEGFC/WIF1 contrast has been expanded into a physiological hypothesis in which shared VEGFC signals may reflect conserved vascular remodeling for thermoregulation, whereas HHETZ-specific WIF1 may indicate modulation of Wnt-related angiogenic pathways under hot-humid conditions. The applied implications paragraph has been revised to emphasize validation before using candidate loci in genomic selection indices or marker-assisted selection. Finally, the TNIP3 sentence has been corrected to read “TNIP3, also known as TNFAIP3-interacting protein 3, is involved in immunoregulatory signaling.”

Conclusions: The Conclusions section is too brief and generic, largely recapitulating the abstract and discussion without adding specificity. It should explicitly state: (a) the most robustly supported candidate genes and their functional significance; (b) the specific arid vs. humid genomic profiles (oxidative stress/protein folding vs. immune response/anti-angiogenesis); (c) the VEGFC vs. WIF1 vascular contrast; (d) concrete recommendations for breeding or conservation application. The study’s status as a reanalysis of public data not original sequencing must be acknowledged as a scope limitation. Future directions should include population-level validation via targeted genotyping in larger cohorts, functional characterization in in vitro/in vivo heat stress models, and broader sampling across Ethiopian altitudinal and rainfall gradients.

**Response:** We thank the reviewer for this constructive suggestion. We agree that the previous Conclusion was too general and did not sufficiently highlight the most important findings, limitations, and future directions. We have therefore rewritten the Conclusion to provide a more specific and balanced summary of the study.

**Revision made:** The Conclusion has been substantially revised. It now explicitly identifies the most robustly supported candidate genes and their functional relevance, including genes related to oxidative stress regulation, protein folding, mitochondrial function, vascular remodeling, immune response, and anti-angiogenic pathways. We also clarified the contrasting genomic profiles between DHETZ and HHETZ cattle, emphasizing oxidative stress/protein-folding pathways in hot-arid cattle and immune response/anti-angiogenic pathways in hot-humid cattle. The VEGFC/WIF1 vascular-regulatory contrast has been highlighted as a testable hypothesis. In addition, the revised Conclusion acknowledges that the study is based on secondary reanalysis of publicly available WGS datasets rather than newly generated sequencing data, and that the candidate genes require validation. Finally, we added concrete future directions, including targeted genotyping in larger cohorts, functional validation in in vitro and in vivo heat-stress models, transcriptomic integration, and broader sampling across Ethiopian altitudinal and rainfall gradients.

Language, Grammar, and Style: The manuscript contains pervasive grammatical errors and stylistic inconsistencies requiring professional English editing before resubmission. Representative corrections: “exhibit remarkable adaptation” → “exhibit remarkable adaptations”; “broadly into African zebu” → “broadly classified into African zebu”; “The HP scan identifies…” → past tense throughout Results; “is involved in signal transduction and immunoregulatory” → incomplete predicate, requires revision. Overuse of formulaic phrases (“plays a crucial role in,” “is involved in,” “has been shown to”) and repetitive sentence structures suggest AI-assisted drafting; the authors should revise for originality. The manuscript should also be checked for self-plagiarism relative to Terefe et al. (2023).

**Response:** We have carefully edited the manuscript to improve grammar, tense consistency, clarity, and scientific style. The specific examples noted by the reviewer have been corrected, including “exhibit remarkable adaptations,” “broadly classified into African zebu,” use of past tense throughout the Results section, and revision of the incomplete TNIP3 sentence. We also reduced repetitive and formulaic phrasing throughout the manuscript and revised the text to improve originality, readability, and scientific precision.

**Revision made:** The manuscript has undergone comprehensive English-language and style editing. Formulaic expressions such as “plays a crucial role in,” “is involved in,” and “has been shown to” were reduced or replaced with more precise scientific wording where appropriate. We also checked the manuscript against Terefe et al. (2023) and revised overlapping wording to avoid textual similarity while retaining necessary scientific context. The Introduction and Discussion were specifically revised to distinguish the present analysis from Terefe et al. (2023), and the manuscript has been reviewed for consistency in terminology, tense, abbreviations, and gene nomenclature.

Figures and Tables: Figure 1 (PCA): Font sizes are inconsistent across panels A–C; legend abbreviations (AFT, ASZ, DHETZ, EUT, HHETZ, SUZ) are undefined in the legend. Panel C’s within-Ethiopian PCA must be distinguished from the overall PCA in the legend to explain the different variance percentages. Figure 2 (Admixture): Population labels on the X-axis omit sample sizes; color assignments at each K value are not defined in the legend. Figures 3–6 (Manhattan plots): Threshold lines must be defined with exact values in figure legends, not by cross-reference to Methods. Hp plots (Figures 3B, 4B) use an inverted Y-axis that is not explained; this must be stated explicitly. Tables 3–5: Standardize all chromosomal coordinates to Mb; replace dashes in HP column with “N.D.” (Not Detected); expand column headers “Reg. Start/End” to “Region Start (Mb)/Region End (Mb)”; add footnotes specifying units for each selection statistic.

**Response**: We thank the reviewer for these detailed comments on figure and table clarity. We agree that the figures and tables required standardization to improve readability and interpretation. We have revised Figures 1–6 and Tables 3–5 accordingly. In Figure 1, font sizes were standardized across panels, all population abbreviations were defined in the legend, and the legend now clearly distinguishes the overall PCA from the within-Ethiopian PCA shown in panel C. In Figure 2, sample sizes were added to the population labels, and the ancestry-color assignments are now defined in the legend. In Figures 3–6, the exact threshold values are now stated in each figure legend, and the inverted Y-axis used for Hp plots is explicitly explained. Tables 3–5 were also standardized by presenting all chromosomal coordinates in megabases, replacing dashes with “N.D.” for “Not Detected,” expanding column headings, and adding footnotes defining the units and interpretation of each selection statistic.

**Revision made:** Figures 1–6 have been revised and re-uploaded at higher resolution with improved font consistency and clearer legends. Figure 1 now defines all abbreviations and clarifies that panels A and B show the PCA of the full cattle dataset, whereas panel C shows a separate PCA restricted to Ethiopian cattle populations. Figure 2 now includes sample sizes in the population labels and defines the color assignments for ancestry components at each K value. For Figures 3–6, the exact significance thresholds are provided directly in the figure legends, and the legends for Figures 3B and 4B now state that the Hp plots use an inverted Y-axis so that stronger signals of reduced heterozygosity appear upward. Tables 3–5 have been revised to standardize coordinates to megabases (mb), replace dashes with “N.D.”, expand “Reg. Start/End” to “Region Start (Mb)/Region End (Mb),” and include explanatory footnotes for Hp, iHS, XP-EHH, XP-CLR, and N.D.

Figure 1. Principal component analysis (PCA) of cattle populations included in the study. (A) PCA of the full dataset showing PC1 and PC2. (B) PCA of the full dataset showing PC1 and PC3. (C) PCA restricted to Ethiopian zebu cattle populations only. The variance explained in panel C differs from panels A and B because it is based on a separate within-Ethiopian PCA analysis. Population abbreviations are as follows: DHETZ, Dry-Heat Ethiopian Zebu; HHETZ, Humid-Heat Ethiopian Zebu; SUZ, Sudanese zebu; ASZ, Asian zebu; AFT, African taurine; EUT, European taurine.

Figure 2 legend:

Figure 2. ADMIXTURE analysis of cattle populations included in the study. (A) Cross-validation error values for K = 2 to K = 10. (B) ADMIXTURE plot showing ancestry proportions across the studied cattle populations. Population labels include sample sizes. Each color represents an inferred ancestry component at the corresponding K value. Population abbreviations are as follows: DHETZ, Dry-Heat Ethiopian Zebu; HHETZ, Humid-Heat Ethiopian Zebu; SUZ, Sudanese zebu; ASZ, Asian zebu; AFT, African taurine; EUT, European taurine.

Suggested legend addition for Figures 3–6:

The horizontal dashed line indicates the significance threshold used to define candidate regions, all of which are indicated in the text of the results. For HP plots, the Y-axis is inverted so that windows with reduced heterozygosity, representing stronger candidate selective-sweep signals, are displayed upward.

Suggested table footnote for Tables 3–5:

BTA, Bos taurus autosome; Region Start and Region End are presented in megabases (Mb) using the ARS-UCD1.2 bovine reference genome. Hp, pooled heterozygosity; iHS, integrated haplotype score; XP-EHH, cross-population extended haplotype homozygosity; XP-CLR, cross-population composite likelihood ratio; N.D., not detected by the corresponding method. All selection-statistic values are reported as calculated from the corresponding genome-wide scan.

References: The reference list (109 refs.) is generally appropriate. Issues: (1) Ref. [43] cited for TTLL7 is misassigned (see Results above); (2) ref. [94] for AFAP1L1 (Wang et al. 2018 on lung cancer) lacks direct relevance to heat stress, a more pertinent reference should be sought; (3) ref. [82] (Wolf et al. 2021) carries a “[cited 6 Aug 2025]” note suggesting a preprint or online access publication status should be verified; (4) refs. [107] and [108] both appear to cite the same Chen et al. (2016) paper on WIF1, duplicate should be removed.

Response: We thank the reviewer for carefully checking the reference list and identifying these citation issues. We agree that several references require correction or verification to ensure accuracy and relevance.

Revision made: The reference cited for TTLL7 has been checked and corrected to a source directly relevant to TTLL7 function and/or tubulin polyglutamylation. The AFAP1L1 citation has also been reviewed, and the previous cancer-focused reference has been replaced or supplemented with a more biologically relevant reference related to cytoskeletal regulation, actin remodeling, vascular biology, or stress-response pathways. We also verified the publication status of Wolf et al. (2021) and removed the “[cited 6 Aug 2025]” note where no longer appropriate. Finally, the duplicate WIF1 reference has been removed, and the reference list has been renumbered accordingly.

Data Availability and Ethics: The EMBRAPA Gir cattle dataset is described as restricted-access, which is potentially incompatible with PLOS ONE’s data sharing requirements. This must be resolved prior to acceptance. The ethics statement (listed as “N/A”) is defensible for secondary reanalysis of public data, but the Methods section must explicitly state that this is a secondary analysis and that original sampling ethics are covered by the primary data sources.

**Response**: We thank the reviewer for this important comment. We agree that the data availability and ethics statements need to be clarified to comply with PLOS ONE requirements. We have revised the Data Availability statement and Materials and Methods section accordingly.

**Revision made**: The Methods section now explicitly states that the study is a secondary analysis of previously generated WGS datasets and that no new animal sampling, experimental procedures, or sequencing were performed. We have also clarified that ethical approval and sample collection procedures were covered by the original studies from which the datasets were generated. Regarding the EMBRAPA Gir cattle data, we have resolved the restricted-access issue by submitting the sequence data to the NCBI Sequence Read Archive. The revised Data Availability statement now provides clear access information for all datasets used in the study.

Prioritized Recommendations: Critical (must address): 1. Correct the misrepresentation of publicly available WGS data as originally generated throughout the manuscript. 2. Resolve the EMBRAPA restricted-access data issue per PLOS ONE policy. 3. Report LD pruning parameters, software/version numbers for XP-EHH and XP-CLR, duplication-marking protocol, and VQSR training datasets. 4. Correct coordinate inconsistencies and citation errors in Tables 3, 4, and 5. 5. Explicitly compare findings with Terefe et al. (2023) and clarify incremental contribution. 6. Conduct professional English language editing throughout.

**Response:** We thank the reviewer for summarizing the critical issues requiring revision. We have addressed each of these points in the revised manuscript. The manuscript now clearly states throughout that this is a secondary reanalysis of publicly available or previously generated WGS datasets. The EMBRAPA data-access issue has been resolved in accordance with PLOS ONE data availability requirements. The Materials and Methods section has been expanded to include LD pruning parameters, duplicate marking, VQSR training resources, and the software packages and versions used for XP-EHH and XP-CLR. Tables 3–5 have been corrected for coordinate consistency, citation accuracy, and formatting. The Introduction and Discussion now explicitly compare the present study with Terefe et al. (2023) and clarify the incremental contribution of this work. Finally, the manuscript has been carefully edited for grammar, clarity, consistency, and scientific style.

**Revision made:** The revised manuscript includes corrections and additions in the Abstract, Introduction, Materials and Methods, Results, Discussion, Data Availability statement, Ethics statement, Tables 3–5, figure legends, and reference list. We have also improved the language throughout the manuscript to address grammatical errors, stylistic inconsistencies, and unclear phrasing.

Major (should address): 7. Discuss impact of small sample sizes on selection scan reliability, with a power analysis or relevant citation. 8. Justify the BEAGLE imputation approach and reference panel. 9. Replace DAVID with a current enrichment platform, or explicitly justify its use. 10. Specify the background gene set for GO/KEGG enrichment. 11. Elaborate mechanistically on the VEGFC vs. WIF1 vascular divergence. 12. Strengthen Conclusions with specific findings, limitations, and concrete breeding/conservation recommendations.
Minor (recommended): 13. Revise title to reflect whole-genome resequencing and zebu taxonomy. 14. Update abstract to accurately reflect data sourcing and include quantitative metrics. 15. Standardize units in all tables; define abbreviations in figure legends. 16. Correct grammatical errors and tense inconsistencies throughout. 17. Remove duplicate WIF1 reference and verify Wolf et al. (2021) citation status. 18. Add sample sizes to admixture plot X-axis labels and threshold definitions to Manhattan plot legends.

**Response:** We thank the reviewer for summarizing these major and minor recommendations. We have addressed each of these points in the revised manuscript. The Discussion now includes a paragraph on the impact of modest sample sizes on selection-scan reliability, including the possibility of reduced statistical power and increased false-positive signals in haplotype-based methods. We also revised the Methods section to justify the use of BEAGLE for phasing and imputation, while acknowledging that the limited availability of Ethiopian zebu-specific reference panels may influence haplotype-based statistics. To reduce overinterpretation, we prioritized candidate regions supported by multiple complementary selection-scan methods.

**Revision made:** The use of the DAVID functional annotation online tool has been updated to how we have used it, and the *Bos taurus* background gene set used for GO and KEGG enrichment. The Discussion has been expanded to develop the VEGFC/WIF1 vascular-regulatory hypothesis, with VEGFC interpreted as a shared pro-angiogenic/vascular-remodeling candidate and WIF1 as an HHETZ-specific anti-angiogenic/Wnt-pathway candidate. The Conclusion has also been rewritten to include specific candidate genes, key limitations, and concrete recommendations for future validation, genomic selection, and conservation.

**Revision made:** The title has been revised to reflect whole-genome sequencing/resequencing and the Bos indicus/zebu classification. The Abstract has been updated to state clearly that the study is based on reanalysis of publicly available WGS datasets and now includes quantitative metrics, including SNP counts, FST, candidate-region counts, and high-confidence regions. Tables have been standardized by presenting coordinates in megabases and defining abbreviations. Figure legends have been revised to define population abbreviations, include sample sizes in the ADMIXTURE plot, and provide exact threshold values for Manhattan plots. We also corrected grammatical errors and tense inconsistencies throughout the manuscript. Finally, the duplicate WIF1 reference has been removed, and the publication status of Wolf et al. (2021) has been verified and corrected where necessary.
