# Supplementary material for: Whole-genome sequencing reveals divergent and shared selection signatures of heat stress adaptation in indigenous Ethiopian zebu cattle from dry-hot and humid-hot environments
Source: PLoS One. 2026 Sep 3;21(9):e0343484. doi: 10.1371/journal.pone.0343484 (PMC13541185; doi:10.1371/journal.pone.0343484)
Supplement: Peer review history

# Peer review: Decision Letter 0

**DOI:** 10.1371/journal.pone.0343484.r001

**Authors:** Manasa Varra

18 May 2026

Dear Dr. Terefe,

Thank you for submitting your manuscript to PLOS ONE. After careful consideration, we feel that it has merit but does not fully meet PLOS ONE’s publication criteria as it currently stands. Therefore, we invite you to submit a revised version of the manuscript that addresses the points raised during the review process.

Please submit your revised manuscript by Jul 02 2026 11:59PM. If you will need more time than this to complete your revisions, please reply to this message or contact the journal office at plosone@plos.org. When you're ready to submit your revision, log on to https://www.editorialmanager.com/pone/ and select the 'Submissions Needing Revision' folder to locate your manuscript file.

- A letter that responds to each point raised by the academic editor and reviewer(s). You should upload this letter as a separate file labeled 'Response to Reviewers'.
- A marked-up copy of your manuscript that highlights changes made to the original version. You should upload this as a separate file labeled 'Revised Manuscript with Track Changes'.
- An unmarked version of your revised paper without tracked changes. You should upload this as a separate file labeled 'Manuscript'.

If you would like to make changes to your financial disclosure, please include your updated statement in your cover letter. Guidelines for resubmitting your figure files are available below the reviewer comments at the end of this letter.

If applicable, we recommend that you deposit your laboratory protocols in protocols.io to enhance the reproducibility of your results. Protocols.io assigns your protocol its own identifier (DOI) so that it can be cited independently in the future. For instructions see: https://journals.plos.org/plosone/s/submission-guidelines#loc-laboratory-protocols. Additionally, PLOS ONE offers an option for publishing peer-reviewed Lab Protocol articles, which describe protocols hosted on protocols.io. Read more information on sharing protocols at https://plos.org/protocols?utm_medium=editorial-email&utm_source=authorletters&utm_campaign=protocols.

As the corresponding author, your ORCID iD is verified in the submission system and will appear in the published article. PLOS supports the use of ORCID, and we encourage all coauthors to register for an ORCID iD and use it as well. Please encourage your coauthors to verify their ORCID iD within the submission system before final acceptance, as unverified ORCID iDs will not appear in the published article. *Only*  the individual author can complete the verification step; PLOS staff *cannot*  verify ORCID iDs on behalf of authors.

We look forward to receiving your revised manuscript.

Kind regards,

Manasa Varra

Academic Editor

PLOS One

**Journal Requirements:**

When submitting your revision, we need you to address these additional requirements.

1. Please ensure that your manuscript meets PLOS ONE's style requirements, including those for file naming. The PLOS ONE style templates can be found at

https://journals.plos.org/plosone/s/file?id=wjVg/PLOSOne_formatting_sample_main_body.pdf and

https://journals.plos.org/plosone/s/file?id=ba62/PLOSOne_formatting_sample_title_authors_affiliations.pdf

2. Please note that PLOS One has specific guidelines on code sharing for submissions in which author-generated code underpins the findings in the manuscript. In these cases, we expect all author-generated code to be made available without restrictions upon publication of the work. Please review our guidelines at https://journals.plos.org/plosone/s/materials-and-software-sharing#loc-sharing-code and ensure that your code is shared in a way that follows best practice and facilitates reproducibility and reuse.

3. Thank you for stating the following financial disclosure:

“The ILRI livestock genetics and genomics program is supported by the One CGIAR SAAF Science Program and its predecessor SAPLING, which is supported by contributors to the CGIAR Trust Fund (http://www.cgiar.org/about-us/our-funders/). This research was funded in part by the Gates Foundation and with UK aid from the UK Foreign, Commonwealth and Development Office (Grant Agreement INV- 040641) under the auspices of the Centre for Tropical Livestock Genetics and Health (CTLGH), established jointly by the University of Edinburgh, SRUC (Scotland's Rural College) and the International Livestock Research Institute (ILRI). The findings and conclusions contained within are those of the authors and do not necessarily reflect the positions or policies of the Gates Foundation or the UK Government. Additionally, this work was funded by the Deanship of Scientific Research at King Faisal University (Research Grant No. KFU253562) and by the Chinese Government's contribution to the CAAS-ILRI Joint Laboratory on Livestock and Forage Genetic Resources in Beijing (2018-GJHZ-01).”

Please state what role the funders took in the study.  If the funders had no role, please state: "The funders had no role in study design, data collection and analysis, decision to publish, or preparation of the manuscript."

If this statement is not correct you must amend it as needed.

Please include this amended Role of Funder statement in your cover letter; we will change the online submission form on your behalf.

4. Thank you for stating the following in the Acknowledgments Section of your manuscript:

“African cattle Genetic Research project for supporting the study 494 and funding a PhD research fellowship.”

We note that you have provided funding information that is not currently declared in your Funding Statement. However, funding information should not appear in the Acknowledgments section or other areas of your manuscript. We will only publish funding information present in the Funding Statement section of the online submission form.

Please remove any funding-related text from the manuscript and let us know how you would like to update your Funding Statement. Currently, your Funding Statement reads as follows:

“The ILRI livestock genetics and genomics program is supported by the One CGIAR SAAF Science Program and its predecessor SAPLING, which is supported by contributors to the CGIAR Trust Fund (http://www.cgiar.org/about-us/our-funders/). This research was funded in part by the Gates Foundation and with UK aid from the UK Foreign, Commonwealth and Development Office (Grant Agreement INV- 040641) under the auspices of the Centre for Tropical Livestock Genetics and Health (CTLGH), established jointly by the University of Edinburgh, SRUC (Scotland's Rural College) and the International Livestock Research Institute (ILRI). The findings and conclusions contained within are those of the authors and do not necessarily reflect the positions or policies of the Gates Foundation or the UK Government. Additionally, this work was funded by the Deanship of Scientific Research at King Faisal University (Research Grant No. KFU253562) and by the Chinese Government's contribution to the CAAS-ILRI Joint Laboratory on Livestock and Forage Genetic Resources in Beijing (2018-GJHZ-01).”

Please include your amended statements within your cover letter; we will change the online submission form on your behalf.

5. You have indicated that data is available from [marcos.vb.silva@embrapa.br]. Please can we ask you to provide us with a general contact email address for the data requests, so readers can request access in perpetuity. If a general email is not available please provide a link to a website where readers can obtain access to data.

6. Please upload a new copy of Figures 1 to 6 as the detail is not clear. Please follow the link for more information:  https://journals.plos.org/plosone/s/figures

7. If the reviewer comments include a recommendation to cite specific previously published works, please review and evaluate these publications to determine whether they are relevant and should be cited. There is no requirement to cite these works unless the editor has indicated otherwise.

**Additional Editor Comments:**

Dear Endashaw Terefe,

The manuscript number PONE-D-26-06464, entitled "The genomic basis of heat stress adaptation in indigenous Ethiopian cattle from arid and hot-humid climates," requires major revision to comply with the standards of PLOS One. Hence, it is recommended for submission in revised form, duly addressing all the reviewer's comments.

Regards,

Dr. Manasa. V

[Note: HTML markup is below. Please do not edit.]

Reviewers' comments:

Reviewer's Responses to Questions

**Comments to the Author**

1. Is the manuscript technically sound, and do the data support the conclusions?

Reviewer #1: Partly

**********

2. Has the statistical analysis been performed appropriately and rigorously?

Reviewer #1: No

**********

3. Have the authors made all data underlying the findings in their manuscript fully available?

The PLOS Data policy

Reviewer #1: No

**********

4. Is the manuscript presented in an intelligible fashion and written in standard English?

Reviewer #1: Yes

**********

Reviewer #1: Section-Wise Evaluation

Title: The title is informative but vague. “Genomic basis” does not distinguish this study from array-based genotyping work, and “indigenous Ethiopian cattle” omits the scientifically important Bos indicus (zebu) classification. A revised title is proposed: “Whole-Genome Sequencing Reveals Divergent and Shared Selection Signatures of Heat Stress Adaptation in Indigenous Ethiopian Zebu Cattle from Hot-Arid and Hot-Humid Environments.” This is more specific, methodologically precise, and avoids implying mechanistic resolution beyond what selection scans can provide.

Abstract: The abstract is generally well-structured but has three key problems. First, it implies that sequencing was conducted as part of this study, whereas all WGS data were retrieved from public repositories (NCBI SRA, CNGB, EMBRAPA). This must be corrected throughout. Second, it lacks quantitative metrics: no FST values are cited for population differentiation, and no counts of candidate regions per method or environment are reported. Third, the concluding novelty statement is generic; a more specific formulation e.g., highlighting the VEGFC vs. WIF1 vascular divergence, would better distinguish the study. Minor language issues (e.g., “remarkably adaptation,” passive-heavy constructions) also require correction.

Introduction: The Introduction is logically organized but does not clearly articulate how this study advances beyond the prior work of Terefe et al. (2023, ref. [11]), which includes some of the same authors and overlapping populations. This distinction is essential. Additional gaps include: (a) the agro-ecological description of hot-arid vs. hot-humid zones is deferred entirely to Methods; a brief overview here would better contextualize the biological rationale; (b) notable mechanisms such as epigenetic regulation, copy number variants (CNVs), and microbiome-mediated thermotolerance are unacknowledged as complementary avenues; (c) the final paragraph describing objectives should include an explicit a priori hypothesis (e.g., whether divergent or partially shared selection is expected given low FST). Language issues: “broadly into African zebu” should read “broadly classified into African zebu”; transition sentences between paragraphs on heat-stress physiology and Ethiopian cattle ecology are abrupt.

Materials and Methods:

Data sourcing: The critical concern is that all WGS data were retrieved from public repositories, yet the abstract and introduction imply original sequencing. This must be corrected throughout for transparency and compliance with PLOS ONE data policies. The inclusion of restricted-access Gir cattle data from EMBRAPA must be resolved, either by excluding these animals and assessing impact, or by obtaining documented permission for release upon acceptance. The HHETZ group has only 18 animals, and some ASZ populations have as few as two individuals per breed. The authors must discuss how these small sample sizes may inflate false-positive rates for iHS and XP-EHH, ideally with a formal power analysis or citation to relevant literature.

Bioinformatics pipeline: Several critical details are absent: (1) duplication marking (e.g., Picard/GATK MarkDuplicates) is not mentioned; (2) sequencing depth per sample and depth cutoffs for filtering are unspecified; (3) VQSR training datasets for bovine WGS are not described, nor are the hard filters applied (QD, FS, MQ, SOR thresholds); (4) LD pruning parameters for ADMIXTURE (window size and step) are incomplete, only the threshold (r² > 0.5) is stated.

Selection scan methods: The use of BEAGLE for phasing/imputation is problematic if the reference panel does not adequately represent Ethiopian zebu populations, which could introduce biases in iHS haplotype scoring. The Hp statistic, originally designed for pooled sequencing, is applied to individual-level WGS data without explanation of adaptation. For XP-EHH and XP-CLR, the sliding window rationale (100 kb window, 50 kb step) is not justified, and the specific software packages and versions used are not cited; making the analysis non-reproducible. The inconsistent significance thresholds between DHETZ and HHETZ for the same statistics are unexplained.

Functional annotation: DAVID has not been updated since 2016; more current platforms (g:Profiler, EnrichR) should be used or DAVID’s limitations explicitly acknowledged. The background gene set for GO/KEGG enrichment is not specified, which significantly affects enrichment outcomes.

Results

Variant discovery and population structure: Table 1 contains a typographic error: SUZ biallelic SNPs are listed as “29,460;635” (semicolon) should be “29,460,635.” The FST analysis states “20 samples per group” were used, but HHETZ has only 18 and DHETZ has 28 animals; any subsampling must be described and its effect discussed. The admixture CV error curve does not show a clear minimum at K=4; rather a gradual decrease followed by a plateau, and actual CV error values should be provided in a supplementary table. The PCA discrepancy between overall variance explained (PC1: 48.7%, PC2: 12.8%) and within-Ethiopian variance (PC1: 11.5%, PC2: 10.5%) is not explained; these represent separate analyses and the distinction should be made explicit.

Selection signatures: Candidate gene tables (3–5) contain errors: (1) the TTLL7 reference in Table 3 (ref. [43], Zhang et al. 2023 on Vav3 in myocardial infarction) is unrelated to polyglutamylation and appears misassigned; (2) Table 5 lists a BTA1 region with start 115.5 Mb and end 11.6 Mb; a physically impossible coordinate; (3) chromosomal coordinates in Table 3 are inconsistently presented in Mb and base pairs and must be standardized. The results do not report method overlap rates (e.g., what proportion of iHS-detected regions were confirmed by XP-EHH or XP-CLR), which would help assess statistical consistency. The detection of WIF1 by all four methods in HHETZ; the most robustly supported finding deserves greater emphasis.

Discussion: The Discussion is the strongest section, effectively integrating candidate gene biology with heat stress physiology (SESN2/antioxidant regulation, GRPEL2/mitochondrial chaperoning, XBP1/UPR, MYD88/innate immunity). However, several gaps remain: (1) No explicit comparison with Terefe et al. (2023) is made, which is a significant analytical omission given the overlapping populations. (2) Alternative explanations for selection signals population bottlenecks, founder effects, background selection are underexplored, particularly important given the low FST (0.0063) between DHETZ and HHETZ. (3) The biologically elegant contrast of VEGFC (pro-angiogenic, shared) vs. WIF1 (anti-angiogenic, HHETZ-specific) deserves a developed physiological hypothesis. (4) Practical implications for genomic selection indices or marker-assisted selection programs are vague; this should be made concrete. (5) The sentence “TNFAIP3 (TNIP3 interacting protein 3) is involved in signal transduction and immunoregulatory” is grammatically incomplete and contains a nomenclature error: TNIP3 is TNFAIP3-interacting protein 3, not TNFAIP3.

Conclusions: The Conclusions section is too brief and generic, largely recapitulating the abstract and discussion without adding specificity. It should explicitly state: (a) the most robustly supported candidate genes and their functional significance; (b) the specific arid vs. humid genomic profiles (oxidative stress/protein folding vs. immune response/anti-angiogenesis); (c) the VEGFC vs. WIF1 vascular contrast; (d) concrete recommendations for breeding or conservation application. The study’s status as a reanalysis of public data not original sequencing must be acknowledged as a scope limitation. Future directions should include population-level validation via targeted genotyping in larger cohorts, functional characterization in in vitro/in vivo heat stress models, and broader sampling across Ethiopian altitudinal and rainfall gradients.

Language, Grammar, and Style: The manuscript contains pervasive grammatical errors and stylistic inconsistencies requiring professional English editing before resubmission. Representative corrections: “exhibit remarkable adaptation” → “exhibit remarkable adaptations”; “broadly into African zebu” → “broadly classified into African zebu”; “The HP scan identifies…” → past tense throughout Results; “is involved in signal transduction and immunoregulatory” → incomplete predicate, requires revision. Overuse of formulaic phrases (“plays a crucial role in,” “is involved in,” “has been shown to”) and repetitive sentence structures suggest AI-assisted drafting; the authors should revise for originality. The manuscript should also be checked for self-plagiarism relative to Terefe et al. (2023).

Figures and Tables: Figure 1 (PCA): Font sizes are inconsistent across panels A–C; legend abbreviations (AFT, ASZ, DHETZ, EUT, HHETZ, SUZ) are undefined in the legend. Panel C’s within-Ethiopian PCA must be distinguished from the overall PCA in the legend to explain the different variance percentages. Figure 2 (Admixture): Population labels on the X-axis omit sample sizes; color assignments at each K value are not defined in the legend. Figures 3–6 (Manhattan plots): Threshold lines must be defined with exact values in figure legends, not by cross-reference to Methods. Hp plots (Figures 3B, 4B) use an inverted Y-axis that is not explained; this must be stated explicitly. Tables 3–5: Standardize all chromosomal coordinates to Mb; replace dashes in HP column with “N.D.” (Not Detected); expand column headers “Reg. Start/End” to “Region Start (Mb)/Region End (Mb)”; add footnotes specifying units for each selection statistic.

References: The reference list (109 refs.) is generally appropriate. Issues: (1) Ref. [43] cited for TTLL7 is misassigned (see Results above); (2) ref. [94] for AFAP1L1 (Wang et al. 2018 on lung cancer) lacks direct relevance to heat stress, a more pertinent reference should be sought; (3) ref. [82] (Wolf et al. 2021) carries a “[cited 6 Aug 2025]” note suggesting a preprint or online access publication status should be verified; (4) refs. [107] and [108] both appear to cite the same Chen et al. (2016) paper on WIF1, duplicate should be removed.

Data Availability and Ethics: The EMBRAPA Gir cattle dataset is described as restricted-access, which is potentially incompatible with PLOS ONE’s data sharing requirements. This must be resolved prior to acceptance. The ethics statement (listed as “N/A”) is defensible for secondary reanalysis of public data, but the Methods section must explicitly state that this is a secondary analysis and that original sampling ethics are covered by the primary data sources.

Prioritized Recommendations: Critical (must address): 1. Correct the misrepresentation of publicly available WGS data as originally generated throughout the manuscript. 2. Resolve the EMBRAPA restricted-access data issue per PLOS ONE policy. 3. Report LD pruning parameters, software/version numbers for XP-EHH and XP-CLR, duplication-marking protocol, and VQSR training datasets. 4. Correct coordinate inconsistencies and citation errors in Tables 3, 4, and 5. 5. Explicitly compare findings with Terefe et al. (2023) and clarify incremental contribution. 6. Conduct professional English language editing throughout.

Major (should address): 7. Discuss impact of small sample sizes on selection scan reliability, with a power analysis or relevant citation. 8. Justify the BEAGLE imputation approach and reference panel. 9. Replace DAVID with a current enrichment platform, or explicitly justify its use. 10. Specify the background gene set for GO/KEGG enrichment. 11. Elaborate mechanistically on the VEGFC vs. WIF1 vascular divergence. 12. Strengthen Conclusions with specific findings, limitations, and concrete breeding/conservation recommendations.

Minor (recommended): 13. Revise title to reflect whole-genome resequencing and zebu taxonomy. 14. Update abstract to accurately reflect data sourcing and include quantitative metrics. 15. Standardize units in all tables; define abbreviations in figure legends. 16. Correct grammatical errors and tense inconsistencies throughout. 17. Remove duplicate WIF1 reference and verify Wolf et al. (2021) citation status. 18. Add sample sizes to admixture plot X-axis labels and threshold definitions to Manhattan plot legends.

**********

what does this mean?). If published, this will include your full peer review and any attached files.

If you choose “no”, your identity will remain anonymous but your review may still be made public.

**Do you want your identity to be public for this peer review?**  For information about this choice, including consent withdrawal, please see our Privacy Policy

Reviewer #1: **Yes:** Prof. (Dr.) Bharat Bhushan

**********

[NOTE: If reviewer comments were submitted as an attachment file, they will be attached to this email and accessible via the submission site. Please log into your account, locate the manuscript record, and check for the action link "View Attachments". If this link does not appear, there are no attachment files.]

To ensure your figures meet our technical requirements, please review our figure guidelines: https://journals.plos.org/plosone/s/figures

You may also use PLOS’s free figure tool, NAAS, to help you prepare publication quality figures: https://journals.plos.org/plosone/s/figures#loc-tools-for-figure-preparation.

NAAS will assess whether your figures meet our technical requirements by comparing each figure against our figure specifications.

Attachment Submitted filename: REVIEW REPORT_peer_review_PONE_D_26_06464 (03-04-2026).docx

# Author comment: Author response to Decision Letter 1

**DOI:** 10.1371/journal.pone.0343484.r002

22 Jul 2026

Response to the reviewer

Title: The genomic basis of heat stress adaptation in indigenous Ethiopian cattle from arid and hot-humid climates

Number: PONE-D-26-06464R1

We would like to express our sincere appreciation for the reviewer’s thorough evaluation of our manuscript. The insights and constructive feedback provided were invaluable in enhancing the quality of our work. We truly commend the reviewer for their diligence and thoughtful suggestions. We have carefully considered each comment and made the necessary revisions to address all the points raised. Thank you once again for the time and effort invested in reviewing our manuscript.

Reviewer #1: Section-Wise Evaluation

Title: The title is informative but vague. “Genomic basis” does not distinguish this study from array-based genotyping work, and “indigenous Ethiopian cattle” omits the scientifically important Bos indicus (zebu) classification. A revised title is proposed: “Whole-Genome Sequencing Reveals Divergent and Shared Selection Signatures of Heat Stress Adaptation in Indigenous Ethiopian Zebu Cattle from Hot-Arid and Hot-Humid Environments.” This is more specific, methodologically precise, and avoids implying mechanistic resolution beyond what selection scans can provide.

Response: The title has been revised on the title page to specify the use of whole-genome sequencing data, the Bos indicus/zebu classification, and the comparative hot-arid versus hot-humid framework.

Abstract: The abstract is generally well-structured but has three key problems. First, it implies that sequencing was conducted as part of this study, whereas all WGS data were retrieved from public repositories (NCBI SRA, CNGB, EMBRAPA). This must be corrected throughout. Second, it lacks quantitative metrics: no FST values are cited for population differentiation, and no counts of candidate regions per method or environment are reported. Third, the concluding novelty statement is generic; a more specific formulation e.g., highlighting the VEGFC vs. WIF1 vascular divergence, would better distinguish the study. Minor language issues (e.g., “remarkably adaptation,” passive-heavy constructions) also require correction.

Response: The Abstract has been revised to clarify that the study is a secondary reanalysis of publicly available whole-genome sequencing datasets rather than newly generated sequencing data. We also added quantitative results, including the number of biallelic autosomal SNPs identified in DHETZ and HHETZ cattle, the low genetic differentiation between DHETZ and HHETZ (FST = 0.0063), the number of candidate regions detected by iHS, Hp, XP-EHH, and XP-CLR, and the number of high-confidence candidate regions supported by multiple selection-scan methods. The concluding sentence was also revised to emphasize the VEGFC/WIF1 vascular-regulatory contrast between arid and humid heat adaptation.

Introduction: The Introduction is logically organized but does not clearly articulate how this study advances beyond the prior work of Terefe et al. (2023, ref. [11]), which includes some of the same authors and overlapping populations. This distinction is essential. Additional gaps include: (a) the agro-ecological description of hot-arid vs. hot-humid zones is deferred entirely to Methods; a brief overview here would better contextualize the biological rationale; (b) notable mechanisms such as epigenetic regulation, copy number variants (CNVs), and microbiome-mediated thermotolerance are unacknowledged as complementary avenues; (c) the final paragraph describing objectives should include an explicit a priori hypothesis (e.g., whether divergent or partially shared selection is expected given low FST). Language issues: “broadly into African zebu” should read “broadly classified into African zebu”; transition sentences between paragraphs on heat-stress physiology and Ethiopian cattle ecology are abrupt.

Materials and Methods:

Response: We thank the reviewer for this detailed and constructive comment. We agree that the Introduction needed to distinguish the present study more clearly from Terefe et al. (2023), provide a stronger biological context for the hot-arid and hot-humid comparison, acknowledge complementary mechanisms of thermotolerance, and present a clearer hypothesis. We have revised the Introduction accordingly.

Revision made: First, we corrected the language in the opening paragraph by replacing “broadly into African zebu” with “broadly classified into African zebu.” Second, we added a transition sentence linking general heat-stress physiology with the specific ecological setting of Ethiopian cattle. Third, we added a brief agro-ecological overview of the hot-arid and hot-humid environments in the introduction, so that the biological rationale is introduced before the detailed description in Materials and Methods. Fourth, we added a new paragraph clarifying how the present study advances beyond Terefe et al. (2023). Specifically, we now state that while Terefe et al. (2023) described population structure, admixture, and genomic diversity in Ethiopian cattle, the present study focuses specifically on shared and environment-specific selection signatures associated with contrasting thermal and ecological pressures. Fifth, we added a sentence acknowledging that epigenetic regulation, copy number variation, and microbiome-mediated mechanisms may also contribute to thermotolerance but are outside the scope of the current SNP-based selection-scan analysis. Finally, we revised the final paragraph of the Introduction to include an explicit a priori hypothesis that, given the low genetic differentiation between DHETZ and HHETZ cattle, these populations would show both shared thermotolerance-related signatures and environment-specific adaptive signals linked to hot-arid versus hot-humid conditions.

Data sourcing: The critical concern is that all WGS data were retrieved from public repositories, yet the abstract and introduction imply original sequencing. This must be corrected throughout for transparency and compliance with PLOS ONE data policies. The inclusion of restricted-access Gir cattle data from EMBRAPA must be resolved, either by excluding these animals and assessing impact, or by obtaining documented permission for release upon acceptance. The HHETZ group has only 18 animals, and some ASZ populations have as few as two individuals per breed. The authors must discuss how these small sample sizes may inflate false-positive rates for iHS and XP-EHH, ideally with a formal power analysis or citation to relevant literature.

Bioinformatics pipeline: Several critical details are absent: (1) duplication marking (e.g., Picard/GATK MarkDuplicates) is not mentioned; (2) sequencing depth per sample and depth cutoffs for filtering are unspecified; (3) VQSR training datasets for bovine WGS are not described, nor are the hard filters applied (QD, FS, MQ, SOR thresholds); (4) LD pruning parameters for ADMIXTURE (window size and step) are incomplete, only the threshold (r² > 0.5) is stated.

Response: We thank the reviewer for highlighting these important concerns regarding data transparency, PLOS ONE data availability requirements, and interpretation of selection-scan results. We agree that the original wording may have unintentionally implied that whole-genome sequencing was newly generated as part of the present study. We have corrected this throughout the manuscript to make clear that this study represents a secondary reanalysis of previously generated whole-genome sequencing datasets obtained from public repositories.

Revision made: The Abstract, Introduction, Materials and Methods, Data Availability statement, Ethics statement, and Conclusion have been revised to state explicitly that no new animal sampling, experimental procedures, or sequencing were performed in the present study. Phrases such as “we performed whole-genome sequencing” and “we analyzed WGS data” have been revised where appropriate to “we reanalyzed publicly available whole-genome sequencing datasets”.

Revision made: A new subsection titled “Data Sources and Ethics Statement” has been added at the beginning of the Materials and Methods section. This subsection clarifies that the study is based on a secondary analysis of previously generated whole genome sequencing (WGS) datasets. It also notes that ethical approval and sample collection procedures were addressed by the original studies that produced these datasets.

Revision made: The Data Availability statement has been revised to clarify the source and accessibility of all whole genome sequencing (WGS) datasets used in this study. To address the issue regarding the restricted-access EMBRAPA Gir cattle dataset, we have obtained permission from Dr. Marcos Vinícius Gualberto Barbosa da Silva (marcos.vb.silva@embrapa.br ), one of the co-authors. He has submitted the WGS data of Gir cattle to the NCBI Sequence Read Archive, and the submission is completed with BioProject accession number PRJNA1493839. The BioProject accession number is included in the Data Availability section and in S1 Table. This clarification has been added to the revised Materials and Methods and Data Availability statements.

Revision made: We have also added a limitations paragraph to the Discussion addressing the modest sample sizes, including the HHETZ group and some ASZ breed subgroups. We now acknowledge that small sample sizes may reduce statistical power and increase the risk of false-positive signals in haplotype-based selection scans such as iHS and XP-EHH. To reduce overinterpretation, we prioritized only candidate regions supported by multiple complementary selection-scan methods and described these loci as putative adaptive candidates requiring validation in larger independent cohorts.

Revision made: Thank you for the suggestions on the Bioinformatics pipeline employed in the analysis, and corrections were done as follows: (1) After the alignment of sequence data to the reference genome (mapping), duplicate reads were identified and marked using the markDuplicate option of Picard software Picard v2.18.2. (2) The Variant Quality Score Recalibration (VQSR) was performed using the GATK VariantRecalibrator, utilizing known variant sources from dbSNP of the Bos taurus reference genome obtained from the Ensembl database (version 150). A tranche sensitivity threshold between 99.9% and 100.0% was applied to assess the accuracy of variant mapping to the reference genome. Ultimately, a truth sensitivity threshold of 99.9% was used to filter the variants. Since we use tranche-sensitive truth filtering, we did not employ hard filtering methods. Besides, all the pipelines we used here were obtained from online sources, which we duly acknowledge.

Selection scan methods: The use of BEAGLE for phasing/imputation is problematic if the reference panel does not adequately represent Ethiopian zebu populations, which could introduce biases in iHS haplotype scoring. The Hp statistic, originally designed for pooled sequencing, is applied to individual-level WGS data without explanation of adaptation. For XP-EHH and XP-CLR, the sliding window rationale (100 kb window, 50 kb step) is not justified, and the specific software packages and versions used are not cited; making the analysis non-reproducible. The inconsistent significance thresholds between DHETZ and HHETZ for the same statistics are unexplained.

Response: We thank the reviewer for this important comment. We agree that the selection-scan methods require additional clarification to improve reproducibility and to avoid overinterpretation of haplotype-based results. We have expanded the Materials and Methods section to provide additional details on phasing/imputation, the use of Hp with individual-level WGS data, the rationale for the sliding-window approach, the software and versions used for XP-EHH and XP-CLR, and the basis for the significance thresholds applied in DHETZ and HHETZ analyses.

Revision made: The “Identification of selection signatures” subsection has been expanded. We now clarify that BEAGLE v5.1 was used for genotype phasing and imputation before iHS analysis, and we acknowledge that the limited availability of Ethiopian zebu-specific reference panels may influence haplotype-based statistics. Therefore, we prioritized candidate regions supported by multiple complementary selection-scan methods rather than relying on iHS alone. We also added an explanation of how Hp was calculated from individual-level WGS data by summarizing heterozygosity across genomic windows, and we clarified that reduced heterozygosity was interpreted as one line of evidence for candidate selective sweeps. In addition, we added the software packages, citations and versions used for XP-EHH and XP-CLR, justified the 100 kb window and 50 kb step size, and explained the empirical/statistical basis for the significance thresholds used in each group. The inconsistent threshold levels in the DHETZ and HHETZ groups arose from the cut point at the top 0.5% of the windows/candidate regions for each selection scan method.

Functional annotation: DAVID has not been updated since 2016; more current platforms (g:Profiler, EnrichR) should be used or DAVID’s limitations explicitly acknowledged. The background gene set for GO/KEGG enrichment is not specified, which significantly affects enrichment outcomes.

Response: We appreciate the reviewer's valuable recommendation. We acknowledge that the functional annotation and enrichment analysis needed further clarification, specifically concerning the enrichment platform and the background gene set, as well as an update on the platform used. We have revised the functional annotation subsection to enhance the reproducibility and interpretation of the enrichment results. The online DAVID bioinformatics tool has been regularly updated, and the current version, which we used to annotate the candidate genes, is 2025 (Sherman et al., 2022) (https://davidbioinformatics.nih.gov/documentation/index.html#release). However, we cited the initially licensed version of the tool (Huang et al., 2009) in the submitted manuscript. Therefore, we have updated the citation to the current version (Sherman et al., 2022). All protein-coding genes identified within the genomic windows from each selection scan analysis were annotated in the ARS-UCD1.2 genome assembly. The Bos taurus genome background was utilized for the functional annotation (GO and KEGG pathways) of these protein-coding genes. Genes that clustered in Gene Ontology (GO) and KEGG pathway terms, with an enrichment fold greater than 1.0 and a Fisher test p-value of less than 0.05, were selected as putative functions for the genes in those clusters.

Results

Variant discovery and population structure: Table 1 contains a typographic error: SUZ biallelic SNPs are listed as “29,460;635” (semicolon) should be “29,460,635.” The FST analysis states “20 samples per group” were used, but HHETZ has only 18 and DHETZ has 28 animals; any subsampling must be described and its effect discussed. The admixture CV error curve does not show a clear minimum at K=4; rather a gradual decrease followed by a plateau, and actual CV error values should be provided in a supplementary table. The PCA discrepancy between overall variance explained (PC1: 48.7%, PC2: 12.8%) and within-Ethiopian variance (PC1: 11.5%, PC2: 10.5%) is not explained; these represent separate analyses and the distinction should be made explicit.

Response: We thank the reviewer for identifying these important issues in the Results section. We have corrected the typographical error in Table 1 and revised the FST description to clarify the exact sample numbers used in the population differentiation analysis. We have also revised the ADMIXTURE results to avoid overstatement of K = 4 as a clear optimum and now describe the cross-validation pattern more cautiously. In addition, the actual cross-validation error values for K = 2 to K = 10 have been added as a supplementary table. Finally, we have clarified that the PCA percentages reported for the full and Ethiopian-only datasets correspond to two separate PCA analyses and are therefore not directly comparable.

Revision made: Table 1 has been corrected by replacing “29,460;635” with “29,460,635.” The FST was calc

Attachment Submitted filename: Response to the reviewer.docx

# Editor report: Decision Letter 1

**DOI:** 10.1371/journal.pone.0343484.r003

**Authors:** Manasa Varra

17 Aug 2026

Whole genomic sequencing reveals divergent and shared selection signatures of heat stress adaptation in indigenous Ethiopian zebu cattle from hot-arid and hot-humid environments.

PONE-D-26-06464R1

Dear Dr. Terefe,

We’re pleased to inform you that your manuscript has been judged scientifically suitable for publication and will be formally accepted for publication once it meets all outstanding technical requirements.

Within one week, you’ll receive an e-mail detailing the required amendments. When these have been addressed, you’ll receive a formal acceptance letter and your manuscript will be scheduled for publication.

An invoice will be generated when your article is formally accepted. Please note, if your institution has a publishing partnership with PLOS and your article meets the relevant criteria, all or part of your publication costs will be covered. Please make sure your user information is up-to-date by logging into Editorial Manager at Editorial Manager® and clicking the ‘Update My Information' link at the top of the page. For questions related to billing, please contact billing support.

If your institution or institutions have a press office, please notify them about your upcoming paper to help maximize its impact. If they’ll be preparing press materials, please inform our press team as soon as possible -- no later than 48 hours after receiving the formal acceptance. Your manuscript will remain under strict press embargo until 2 pm Eastern Time on the date of publication. For more information, please contact onepress@plos.org.

Kind regards,

Manasa Varra

Academic Editor

PLOS One

Additional Editor Comments (optional):

Dear Endashaw Terefe,

I would like to inform that the revised manuscript PONE-D-26-06464R1 entitled "Whole genomic sequencing reveals divergent and shared selection signatures of heat stress adaptation in indigenous Ethiopian zebu cattle from hot-arid and hot-humid environments, is hereby recommended for acceptance for publication in PLOS One.

Reviewers' comments:

# Editor report: Acceptance letter

**DOI:** 10.1371/journal.pone.0343484.r004

**Authors:** Manasa Varra

PONE-D-26-06464R1

PLOS One

Dear Dr. Terefe,

I'm pleased to inform you that your manuscript has been deemed suitable for publication in PLOS One. Congratulations! Your manuscript is now being handed over to our production team.

At this stage, our production department will prepare your paper for publication. This includes ensuring the following:

* All references, tables, and figures are properly cited

* All relevant supporting information is included in the manuscript submission,

* There are no issues that prevent the paper from being properly typeset

You will receive further instructions from the production team, including instructions on how to review your proof when it is ready. Please keep in mind that we are working through a large volume of accepted articles, so please give us a few days to review your paper and let you know the next and final steps.

Lastly, if your institution or institutions have a press office, please let them know about your upcoming paper now to help maximize its impact. If they'll be preparing press materials, please inform our press team within the next 48 hours. Your manuscript will remain under strict press embargo until 2 pm Eastern Time on the date of publication. For more information, please contact onepress@plos.org.

You will receive an invoice from PLOS for your publication fee after your manuscript has reached the completed accept phase. If you receive an email requesting payment before acceptance or for any other service, this may be a phishing scheme. Learn how to identify phishing emails and protect your accounts at https://explore.plos.org/phishing.

If we can help with anything else, please email us at customercare@plos.org.

Thank you for submitting your work to PLOS One and supporting open access.

Kind regards,

PLOS One Editorial Office Staff

on behalf of

Dr. Manasa Varra

Academic Editor

PLOS One
